# Long-term trends in the burden of colorectal cancer in Europe over three decades: a joinpoint regression and age-period-cohort analysis

**DOI:** 10.3389/fonc.2023.1287653

**Published:** 2023-12-05

**Authors:** Dan Long, Chenhan Mao, Zhensheng Zhang, Yaxuan Liu, Jinru Li, Yin Xu, Ying Zhu

**Affiliations:** ^1^ The First Hospital of Hunan University of Chinese Medicine, Changsha, Hunan, China; ^2^ Affiliated Hospital of Integrated Traditional Chinese and Western Medicine, Nanjing University of Chinese Medicine, Nanjing, Jiangsu, China; ^3^ Department of Oncology, The First Traditional Chinese Medicine Hospital of Zhanjiang City, Zhanjiang, Guangdong, China

**Keywords:** colorectal cancer, burden of disease, GBD, joinpoint regression analysis, age-period-cohort analysis, Europe

## Abstract

**Background:**

The burden of colorectal cancer (CRC) in Europe is at a high level, but the epidemiological features have not yet been systematically studied. This study aimed to provide a timely and reliable assessment of the burden and trends of CRC in Europe to provide a scientific basis for its prevention and treatment.

**Methods:**

We analyzed data on CRC in 44 European countries between 1990 and 2019 from the Global Burden of Disease study (GBD) 2019. In addition, the joinpoint regression model was applied to reflect temporal trends. The age-period-cohort model was constructed to explore age, period, and birth cohort effects that influence the risk of morbidity and mortality.

**Results:**

In Europe, new cases, disability-adjusted life years (DALYs) and deaths of CRC rose by 70.01%, 22.88% and 38.04% from 1990 to 2019, respectively. The age-standardized incidence rate (ASIR) has increased, while age-standardized DALY rate and age-standardized mortality rate (ASMR) have declined. We found that men experienced a significantly higher CRC burden than women. Age-period-cohort analysis showed that the risk of incidence and mortality increased with age and time; and it was lower in the later-born cohort than the earlier-born cohort.

**Conclusion:**

ASIR for CRC in Europe generally trended upwards from 1990 to 2019, stabilizing in recent years but still at a high level. CRC burden varied considerably in different countries. There was a pronounced gender difference in CRC burden, and middle-aged and older men should be a priority population for CRC prevention and treatment.

## Introduction

CRC is the third most prevalent cancer in the world and the second leading cause of cancer-related deaths (1). It is estimated that there are approximately 900,000 CRC deaths per year worldwide (1). There are significant regional differences in the disease burden of CRC, with the highest morbidity and mortality rates usually found in countries with a high Human Development Index, such as Europe, Oceania, and North America (2, 3). The economic burden of CRC across Europe in 2015 was €19.1 billion (4). Nevertheless, there is a lack of systematic studies on the epidemiology of CRC in European countries.

GBD 2019 is a substantial database researched by multi-country cooperation that covers all WHO member countries. It provides a comprehensive assessment of health losses due to 369 diseases and injuries and 87 risk factors in 204 countries and territories worldwide between 1990 and 2019 (5). It is currently the most extensive and credible database on the burden of disease worldwide and has been widely used in disease burden studies (5–7). Therefore, in this study, we aimed to evaluate the disease burden and trends of CRC in Europe in a timely and reliable manner, thus providing a scientific basis of some value for CRC prevention and treatment.

## Materials and methods

### Data resource

Data available for this study include cases and ASRs of CRC incidence, mortality, DALY, and age-specific incidence and mortality rates in Europe (comprising 44 countries) and corresponding demographic data. The GBD project is an international collaborative health science research project conducted by the Institute for Health Metrics and Evaluation (IHME) at the University of Washington in conjunction with the World Health Organization (WHO), the World Bank, and the Harvard School of Public Health (8). To provide estimates on the burden of CRC, data were collected using various sources such as vital registration, verbal autopsy, and cancer registries. All GBD estimates in this study were provided with 95% uncertainty intervals (UIs). For each computational step, 1000 draws were generated; 95% UIs were calculated by taking values at the 2.5th and 97.5th percentile from the 1000 draws, and were provided with the mean estimates (9). All data for this study were obtained from the GBD 2019, which can be retrieved via a website tool (https://vizhub.healthdata.org/gbd-results/). The parameters were set as follows: GBD Estimate - Cause of death or injury; Measure - Deaths, DALYs, Incidence; Metric - Number, Rate; Cause - Colon and rectum cancer; Location - Europe, all European countries; Age - Age-standardized, All ages, age groups (5-9 years, 10-14 years,……90-94 years and 95 + years); Sex - Both, Female, Male; Year - from 1990 to 2019. Detailed descriptions of the raw data and general methodology of the GBD 2019 study have been described in previous publications (10).

### Joinpoint regression analysis

To assess trends in ASRs of CRC in Europe between 1990 and 2019, we performed joinpoint regression analysis on different gender populations. Joinpoint regression, also known as segmented regression, is based on the premise that the long-term trendline is divided into segments, each of which is described by a continuous log-linear model (11). The Joinpoint software (version 5.0.2; National Cancer Institute, Rockville, MD, US) was used to understand temporal trends in a structured way and to test which trends between joinpoints were statistically significant (12). A maximum of 6 line segments (5 joinpoints) were applied in the model. AAPC, APC, and corresponding 95% Confidence Interval (CI) were calculated for this study. An APC of >0 for a given stage indicates an increasing trend in morbidity/mortality/DALY rates. Conversely, an APC of <0 for a stage represents a decreasing trend in morbidity/mortality/DALY rates (13). AAPC is the average APC over the entire period considered.

### Age-period-cohort analysis

The APC model is based on a multiple regression model with a Poisson distribution as the essence and is suitable for studies of cancer incidence or mortality (14, 15). In contrast to traditional methods, the APC model can be fitted to the data by controlling for the three factors (age, period, and cohort) of the interaction. Standard regression modelling techniques are not appropriate because of the lack of linear independence between variables where cohort=period-age. As a result, an APC model using the intrinsic estimator (IE) was created for each outcome variable. The result is compelling and unique, with the advantages of no restriction assumption and a wide application range, which became one of the APC model research hotspots. Compared with the traditional generalized linear model, which assumes that two or more coefficients of a parameter vector are equal, the IE algorithm limits the geometric orientation of the parameter vector in the parameter space (16). In the IE method of the APC model, the fixed 5-years group form for age, period and cohort is usually required (6, 17). Accordingly, we used 5 consecutive years as a period/cohort (cohort = period - age) and 5 consecutive years as an age group in this study. Effect coefficient and relative risk (RR) for age, period, and cohort for CRC morbidity and mortality were calculated using Stata 16.0 based on the age-period-cohort model and the IE method. RR = exp (effect coefficient), with larger RR values indicating a higher risk of incidence or mortality.RR values above 1 indicate a higher risk of incidence or mortality relative to the average. Conversely, RR values less than 1 suggest a lower risk of incidence or mortality relative to the average (18).

In addition, R 4.3.1 was used for data analysis and plotting in this study. A *P*-value <0.05 was considered statistically significant.

## Results

### Temporal patterns of CRC burden in Europe

There were 590,376 (95% uncertainty interval (UI): 529,705 to 651,670) new cases of CRC in Europe in 2019 (**Table 1**), an increase of 70.01% compared to 1990 (347,261 (335,664 to 355,050)). In the same year, 5,762,063 (5,402,969 to 6,091,773) DALYs and 298,983 (274,675 to 316,530) deaths were recorded, representing a rise of 22.88% and 38.04%, respectively, compared to 1990.

**Table 1:** CRC incidence, DALY and mortality in Europe in 1990 and 2019 and AAPC from 1990 to 2019.

	1990		2019		1990-2019
	Cases NO.(95%UI)	ASR/100,000 (95% UI)	Cases NO.(95%UI)	ASR/100,000 (95% UI)	AAPC (95%CI)
Incidence Sex					
Both	347260.56 (335664.4 to 355050.09)	33.49 (32.33 to 34.26)	590376.19 (529705.15 to 651670.25)	38.38 (34.56 to 42.47)	0.49 (0.17 to 0.80)
Female	178614.56 (170480.56 to 184119.61)	28.62 (27.38 to 29.5)	268402.31 (237200.69 to 298400.08)	30.43 (27.19 to 33.77)	0.22 (-0.08 to 0.51)
Male	168646.00 (164524.35 to 171973.71)	41.23 (40.06 to 42.09)	321973.88 (289417.17 to 357024.34)	48.88 (44.03 to 54.2)	0.60 (0.28 to 0.92)
DALY Sex					
Both	4689192.52 (4565695.4 to 4801932.34)	458.43 (446.08 to 469.69)	5762062.89 (5402969.47 to 6091772.8)	391.65 (367.89 to 414.26)	-0.54 (-0.79 to -0.29)
Female	2315406.16 (2230570.04 to 2389935.27)	387.68 (374.63 to 400.58)	2543802.53 (2340778.17 to 2721258.56)	307.1 (285.79 to 328.14)	-0.81 (-1.06 to -0.56)
Male	2373786.36 (2320186.82 to 2423249.47)	562.18 (548.5 to 574.56)	3218260.36 (3020619.2 to 3407979.03)	497.92 (467.8 to 527.39)	-0.40 (-0.64 to -0.16)
Mortality Sex					
Both	216586.73 (207793.04 to 222141.61)	21.02 (20.11 to 21.59)	298982.75 (274675.49 to 316529.96)	18.44 (17.07 to 19.50)	-0.44 (-0.67 to -0.22)
Female	113941.44 (107491.58 to 118007.46)	17.88 (16.9 to 18.55)	142087.43 (126946.16 to 152094.71)	14.63 (13.32 to 15.62)	-0.69 (-0.92 to -0.46)
Male	102645.29 (99942.38 to 104750.69)	26.21 (25.33 to 26.79)	156895.32 (145832.77 to 165963.43)	23.69 (22 to 25.07)	-0.34 (-0.59 to -0.09)

**Figure 1:**
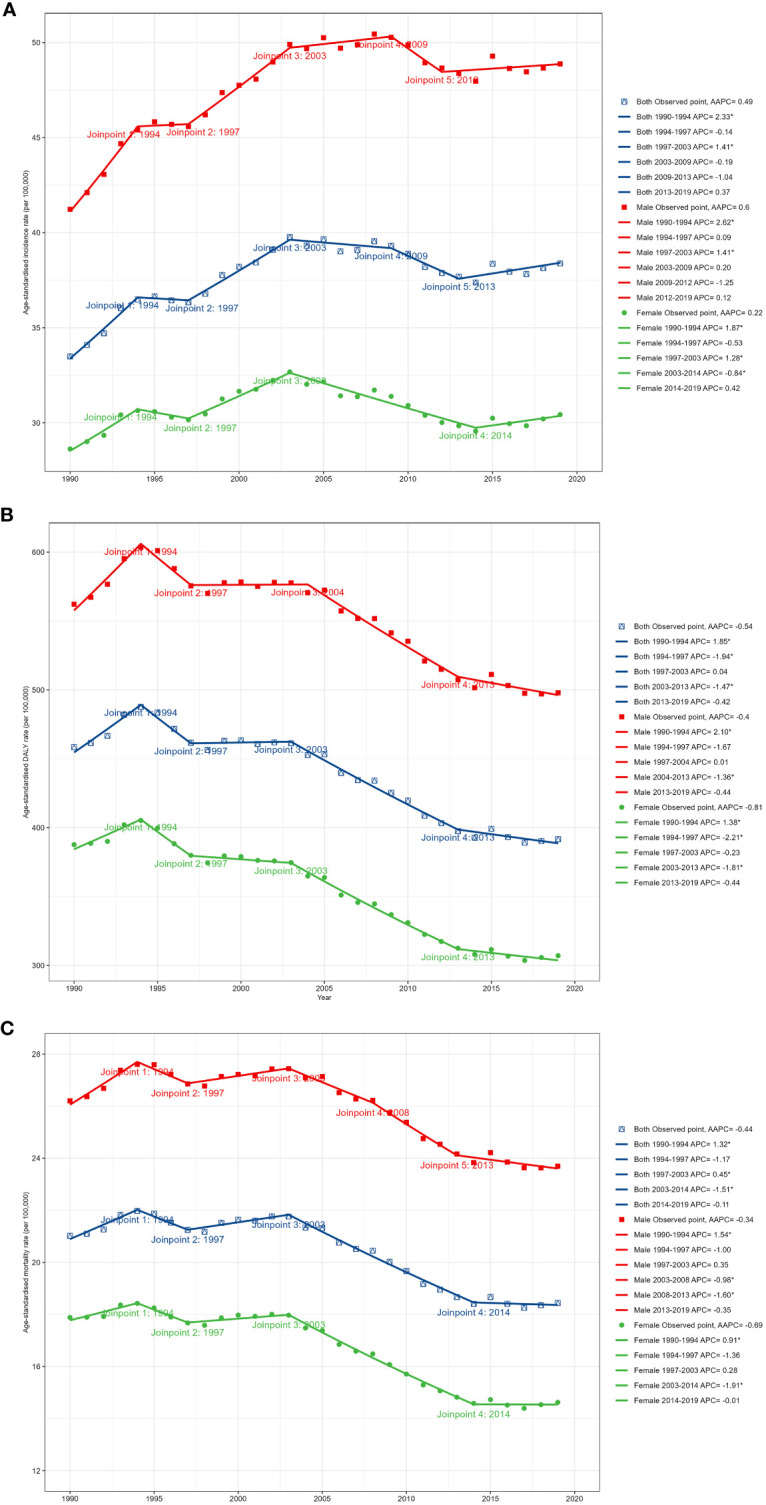
Temporal trends in CRC burden in Europe between 1990 and 2019. **(A)** The age-standardized incidence rate (ASIR). **(B)** The age-standardized prevalence rate (ASPR). **(C)** The age-standardized disability-adjusted life year (DALY) rate.

**Figure 1** and **Table 2** present the joinpoint analysis of trends in ASRs for European CRC from 1990 to 2019. Jointpoint regression results showed that ASIR increased from 33.49 (95% UI: 32.33 to 34.26) per 100,000 population in 1990 to 38.38 (34.56 to 42.47) per 100,000 population in 2019 in both sexes, with an AAPC of 0.49% (95% CI: 0.17% to 0.80%, *P* < 0.05). More specifically, ASIR rose most significantly from 1990 to 1994 (APC: 2.33% (1.72% to 2.94%), *P* < 0.05). From 1994 to 1997 there was a slight but not statistically significant downward trend (APC: -0.14% (-1.97% to 1.72%), *P* > 0.05). From 1997 to 2003 there was another increase (APC: 1.41% (0.93% to 1.89%), *P* < 0.05). From 2003 to 2009 there was a downward trend but not statistically significant (APC: -0.19% (-0.71% to 0.34%), *P* > 0.05). From 2009 to 2013 there was a decrease but not statistically significant (APC: -1.04% (-2.27% to 0.20%), *P* > 0.05). From 2013 to 2019 there was again an upward trend, but not statistically significant (APC: 0.37% (-0.37% to 1.11%), *P* > 0.05).

**Table 2:** Joinpoint regression analysis of ASRs for CRC in Europe from 1990 to 2019.

ASIR			
Sex	Year	P.Value	APC_95%CI
Both	1990-1994	0.00	2.33 (1.72 to 2.94)
Both	1994-1997	0.87	-0.14 (-1.97 to 1.72)
Both	1997-2003	0.00	1.41 (0.93 to 1.89)
Both	2003-2009	0.45	-0.19 (-0.71 to 0.34)
Both	2009-2013	0.09	-1.04 (-2.27 to 0.20)
Both	2013-2019	0.30	0.37 (-0.37 to 1.11)
Female	1990-1994	0.00	1.87 (1.2 to 2.54)
Female	1994-1997	0.60	-0.53 (-2.6 to 1.59)
Female	1997-2003	0.00	1.28 (0.75 to 1.81)
Female	2003-2014	0.00	-0.84 (-1.05 to -0.63)
Female	2014-2019	0.39	0.42 (-0.58 to 1.42)
Male	1990-1994	0.00	2.62 (2.07 to 3.18)
Male	1994-1997	0.91	0.09 (-1.57 to 1.77)
Male	1997-2003	0.00	1.41 (1 to 1.83)
Male	2003-2009	0.38	0.2 (-0.27 to 0.67)
Male	2009-2012	0.24	-1.25 (-3.39 to 0.94)
Male	2012-2019	0.65	0.12 (-0.43 to 0.68)
Age-standardized DALY rate			
Both	1990-1994	0.00	1.85 (1.22 to 2.48)
Both	1994-1997	0.04	-1.94 (-3.75 to -0.10)
Both	1997-2003	0.85	0.04 (-0.42 to 0.50)
Both	2003-2013	0.00	-1.47 (-1.69 to -1.26)
Both	2013-2019	0.16	-0.42 (-1.02 to 0.18)
Female	1990-1994	0.00	1.38 (0.72 to 2.04)
Female	1994-1997	0.02	-2.21 (-4.02 to -0.37)
Female	1997-2003	0.31	-0.23 (-0.69 to 0.24)
Female	2003-2013	0.00	-1.81 (-2.03 to -1.59)
Female	2013-2019	0.14	-0.44 (-1.03 to 0.15)
Male	1990-1994	0.00	2.10 (1.49 to 2.72)
Male	1994-1997	0.07	-1.67 (-3.44 to 0.13)
Male	1997-2004	0.95	0.01 (-0.31 to 0.33)
Male	2004-2013	0.00	-1.36 (-1.6 to -1.12)
Male	2013-2019	0.15	-0.44 (-1.05 to 0.17)
ASMR			
Both	1990-1994	0.00	1.32 (0.79 to 1.85)
Both	1994-1997	0.15	-1.17 (-2.8 to 0.48)
Both	1997-2003	0.04	0.45 (0.03 to 0.87)
Both	2003-2014	0.00	-1.51 (-1.68 to -1.35)
Both	2014-2019	0.73	-0.11 (-0.73 to 0.52)
Female	1990-1994	0.00	0.91 (0.34 to 1.50)
Female	1994-1997	0.12	-1.36 (-3.1 to 0.4)
Female	1997-2003	0.20	0.28 (-0.16 to 0.72)
Female	2003-2014	0.00	-1.91 (-2.09 to -1.73)
Female	2014-2019	0.96	-0.01 (-0.65 to 0.63)
Male	1990-1994	0.00	1.54 (1.03 to 2.05)
Male	1994-1997	0.19	-1 (-2.53 to 0.55)
Male	1997-2003	0.08	0.35 (-0.04 to 0.75)
Male	2003-2008	0.01	-0.98 (-1.61 to -0.34)
Male	2008-2013	0.00	-1.6 (-2.26 to -0.93)
Male	2013-2019	0.14	-0.35 (-0.85 to 0.14)

In contrast, the age-standardized DALY rate and ASMR have declined over the past 30 years. The age-standardized DALY rate decreased from 458.43 (95% UI: 446.08 to 469.69) per 100,000 in 1990 to 391.65 (367.89 to 414.26) per 100,000 in 2019, with an AAPC of -0.54% (95% CI: -0.79% to -0.29%, *P* < 0.05). The ASMR declined from 21.02 (95% UI: 20.11 to 21.59) per 100,000 in 1990 to 18.44 (17.07 to 19.50) per 100,000 in 2019, with an AAPC of -0.44% (95% CI: -0.67% to -0.22%, *P* < 0.05). The most significant decline in the DALY rate occurred from 2003 to 2013 (APC: -1.47% (-1.69% to -1.26%), *P* < 0.05). ASMR increased significantly from 1990 to 1994 (APC: 1.32% (0.79% to 1.85%), *P* < 0.05). There was a downward trend from 1994 to 1997, but not statistically significant (APC: -1.17% (-2.80% to 0.48%), *P* > 0.05). There was an increase from 1997 to 2003 (APC: 0.45% (0.03% to 0.87%), *P* < 0.05). From 2003 to 2014 there was a significant decrease (APC: -1.51% (-1.68% to -1.35%), *P* < 0.05). There was a slight but not statistically significant decrease from 2014 to 2019 (APC: -0.11% (-0.73% to 0.52%), *P* > 0.05).

### Gender differences in CRC

In 2019, men accounted for 54.54% of new CRC cases in Europe. CRC DALYs and deaths followed a similar pattern, with 55.85% of CRC DALYs and 52.48% of deaths belonging to men. Similar gender differences existed for ASRs, as shown in **Figure 1**. From 1990 to 2019, ASIR was significantly higher in men than in women, as were age-standardized DALY rate and ASMR. ASIR for CRC increased in men during the study period (AAPC: 0.60% (95% CI: 0.28 to 0.92), *P* < 0.05), whereas there was no significant trend in women (0.22% (-0.08% to 0.51%), *P* > 0.05). Furthermore, although DALY and mortality rates decreased in both men and women, the decrease was significantly smaller in men than in women. It can be seen that the CRC burden was significantly higher in European men than in women.

### Age-period-cohort analysis

#### Age effect

Smaller values for Akaike’s information criterion (AIC) and Bayesian information criterion (BIC) with parameter penalty terms denote a better fit. For the incidence model, the BIC values for overall gender, male and female were 417.95, -55.89 and 197.96, respectively. The AIC values for both gender, male and female were 17.86, 12,99 and 15.21, respectively, which indicated a good model fit.

**Figure 2:**
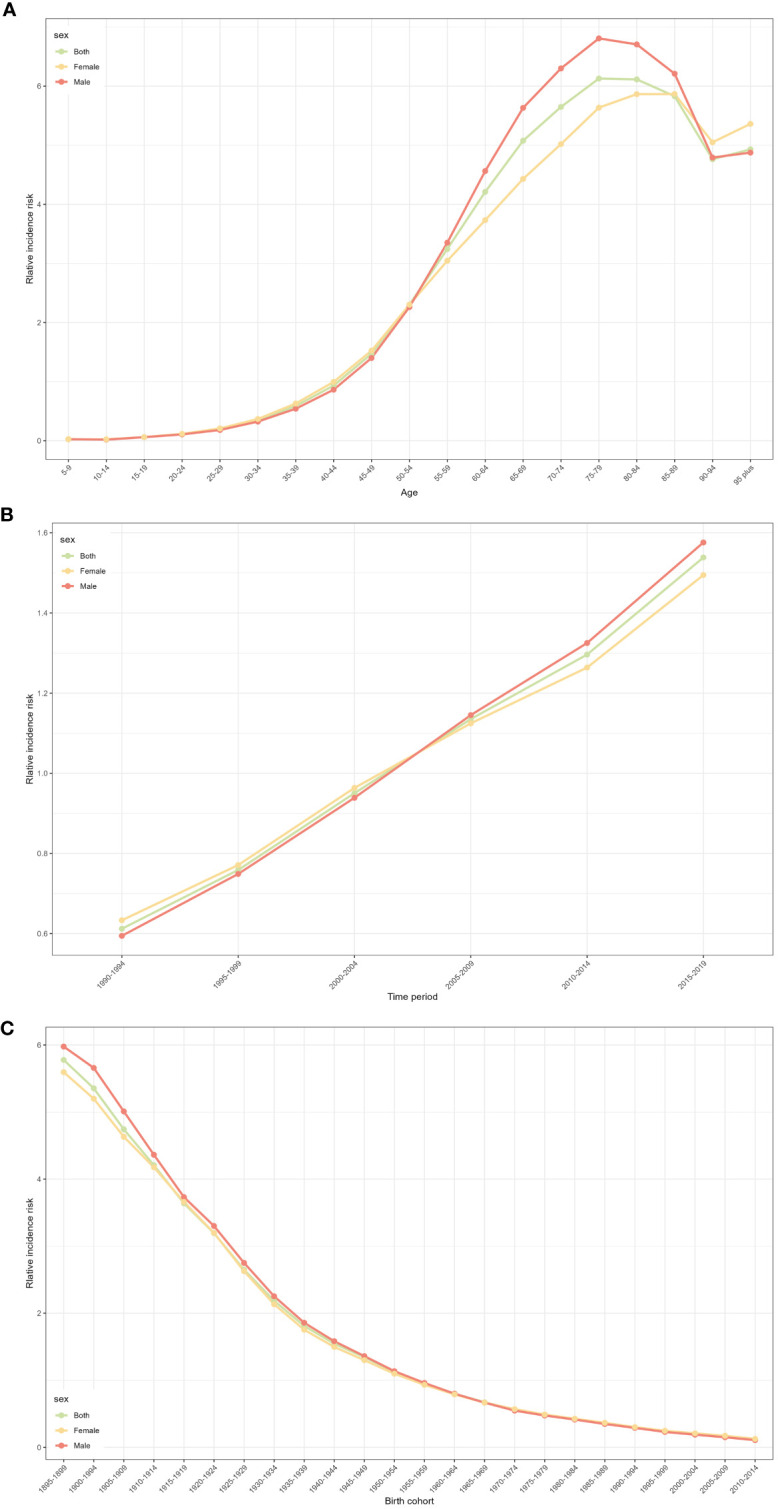
CRC relative incidence risk for male and females due to age, period, and cohort effects in Europe. **(A)** Relative incidence risk due to age effect. **(B)** Relative incidence risk due to period effect. **(C)** Relative incidence risk due to cohort effect.

**Figure 3:**
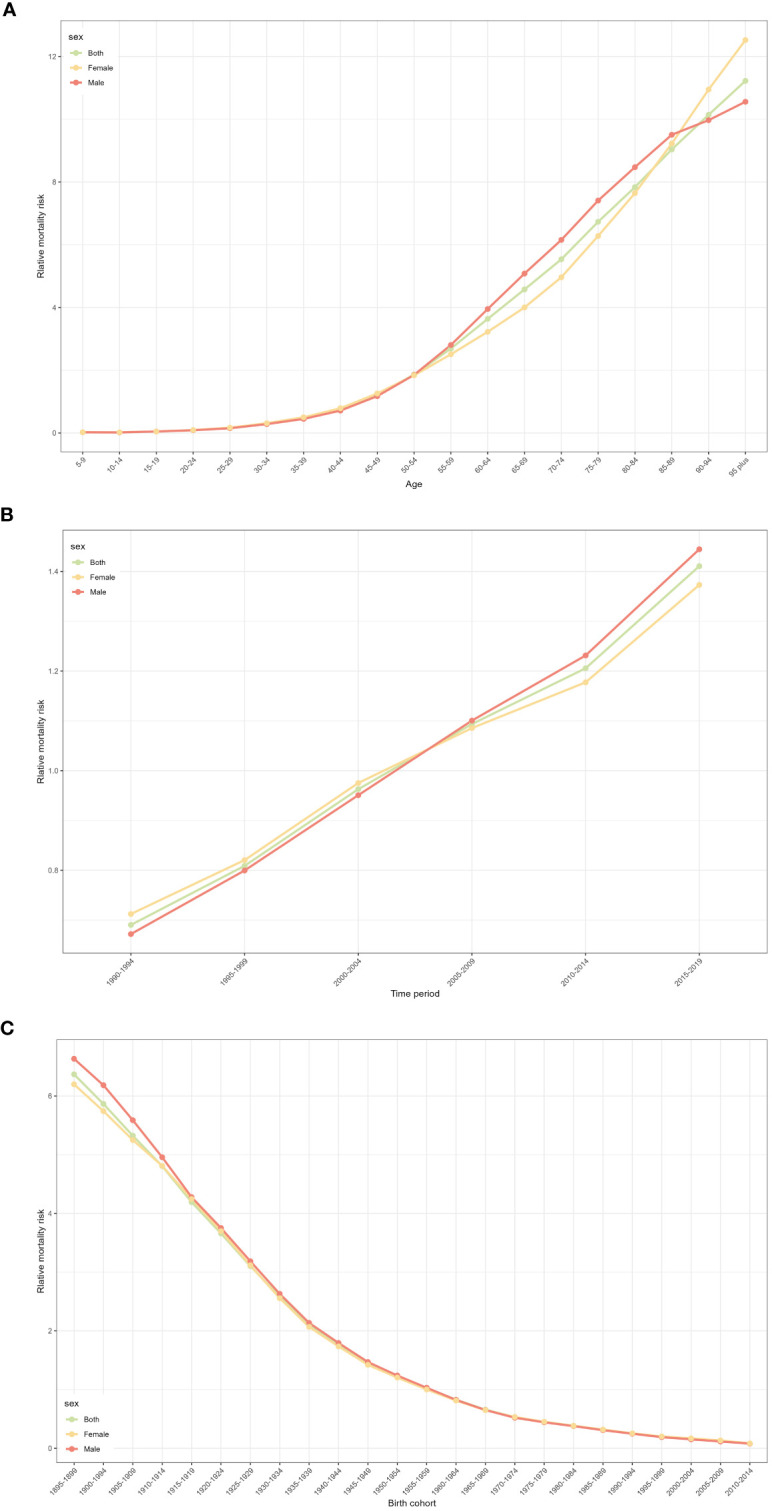
CRC relative mortality risk for male and females due to age, period, and cohort effects in Europe. **(A)** Relative mortality risk due to age effect. **(B)** Relative mortality risk due to period effect. **(C)** Relative mortality risk due to cohort effect.

**Figure 2A** shows the relative incidence risk of CRC by gender. As can be seen in **Figure 2A**, the highest incidence risk was reached in males aged 75-79 years and females aged 85-89 years. In other words, the relative incidence risk of CRC increased with age in both females and males, but began to decline in the 85-89 age group for females and the 75-79 age group for males. The relative incidence risk increased in the 95-plus age group for both males and females. Females aged 50-95 plus years and males aged 45-95 plus years were the two risk groups with a relative incidence risk >1 (**Table 3**). **Figure 3A** shows the relative death risk of CRC by sex. The relative death risk for CRC increased with age for both women and men, with the highest risk of death in those aged 95 years and over. Men and women aged 45-95 plus years were the two risk groups with a relative death risk of >1 (**Table 4**).

**Table 3:** CRC relative incidence risk due to age, period, and cohort effects.

Variables	Both		Male		Female	
	Coef_95%CI	RR_95%CI	Coef_95%CI	RR_95%CI	Coef_95%CI	RR_95%CI
Age						
5-9	-3.64(-3.75 to -3.54)	0.03(0.02 to 0.03)	-3.66(-3.8 to -3.52)	0.03(0.02 to 0.03)	-3.65(-3.8 to -3.5)	0.03(0.02 to 0.03)
10-14	-3.85(-3.94 to -3.75)	0.02(0.02 to 0.02)	-3.97(-4.1 to -3.84)	0.02(0.02 to 0.02)	-3.73(-3.87 to -3.6)	0.02(0.02 to 0.03)
15-19	-2.78(-2.83 to -2.72)	0.06(0.06 to 0.07)	-2.8(-2.87 to -2.73)	0.06(0.06 to 0.07)	-2.79(-2.87 to -2.71)	0.06(0.06 to 0.07)
20-24	-2.18(-2.22 to -2.14)	0.11(0.11 to 0.12)	-2.23(-2.29 to -2.18)	0.11(0.1 to 0.11)	-2.15(-2.21 to -2.09)	0.12(0.11 to 0.12)
25-29	-1.63(-1.66 to -1.6)	0.2(0.19 to 0.2)	-1.7(-1.74 to -1.66)	0.18(0.17 to 0.19)	-1.57(-1.62 to -1.52)	0.21(0.2 to 0.22)
30-34	-1.06(-1.08 to -1.03)	0.35(0.34 to 0.36)	-1.13(-1.16 to -1.09)	0.32(0.31 to 0.34)	-1(-1.04 to -0.96)	0.37(0.35 to 0.38)
35-39	-0.53(-0.55 to -0.51)	0.59(0.57 to 0.6)	-0.61(-0.64 to -0.58)	0.54(0.53 to 0.56)	-0.46(-0.49 to -0.43)	0.63(0.61 to 0.65)
40-44	-0.07(-0.09 to -0.05)	0.93(0.92 to 0.95)	-0.15(-0.17 to -0.12)	0.86(0.84 to 0.89)	0(-0.03 to 0.02)	1.00(0.97 to 1.02)
45-49	0.39(0.37 to 0.4)	1.47(1.45 to 1.5)	0.34(0.31 to 0.36)	1.4(1.37 to 1.43)	0.42(0.4 to 0.44)	1.52(1.49 to 1.56)
50-54	0.83(0.82 to 0.85)	2.30(2.27 to 2.34)	0.82(0.8 to 0.83)	2.26(2.22 to 2.3)	0.83(0.81 to 0.85)	2.3(2.26 to 2.35)
55-59	1.18(1.17 to 1.19)	3.24(3.21 to 3.28)	1.21(1.19 to 1.22)	3.35(3.3 to 3.4)	1.11(1.1 to 1.13)	3.05(3 to 3.09)
60-64	1.44(1.43 to 1.45)	4.21(4.17 to 4.25)	1.52(1.51 to 1.53)	4.56(4.51 to 4.62)	1.32(1.3 to 1.33)	3.73(3.68 to 3.78)
65-69	1.62(1.62 to 1.63)	5.08(5.04 to 5.12)	1.73(1.72 to 1.74)	5.63(5.57 to 5.7)	1.49(1.48 to 1.5)	4.43(4.38 to 4.48)
70-74	1.73(1.72 to 1.74)	5.65(5.6 to 5.69)	1.84(1.83 to 1.85)	6.3(6.23 to 6.37)	1.61(1.6 to 1.63)	5.02(4.96 to 5.08)
75-79	1.81(1.8 to 1.82)	6.13(6.07 to 6.19)	1.92(1.9 to 1.93)	6.81(6.72 to 6.9)	1.73(1.72 to 1.74)	5.64(5.56 to 5.71)
80-84	1.81(1.8 to 1.82)	6.11(6.05 to 6.18)	1.9(1.89 to 1.92)	6.71(6.6 to 6.82)	1.77(1.75 to 1.79)	5.86(5.77 to 5.96)
85-89	1.76(1.75 to 1.78)	5.83(5.75 to 5.91)	1.83(1.81 to 1.85)	6.21(6.09 to 6.33)	1.77(1.75 to 1.79)	5.87(5.75 to 5.98)
90-94	1.56(1.54 to 1.58)	4.76(4.68 to 4.84)	1.57(1.54 to 1.59)	4.79(4.67 to 4.91)	1.62(1.6 to 1.64)	5.05(4.93 to 5.17)
95 plus	1.6(1.57 to 1.62)	4.93(4.82 to 5.04)	1.58(1.55 to 1.62)	4.88(4.71 to 5.05)	1.68(1.65 to 1.71)	5.36(5.2 to 5.53)
Period						
1990-1994	-0.49(-0.5 to -0.48)	0.61(0.61 to 0.62)	-0.52(-0.53 to -0.51)	0.59(0.59 to 0.6)	-0.46(-0.47 to -0.44)	0.63(0.63 to 0.64)
1995-1999	-0.28(-0.28 to -0.27)	0.76(0.75 to 0.76)	-0.29(-0.3 to -0.28)	0.75(0.74 to 0.75)	-0.26(-0.27 to -0.25)	0.77(0.77 to 0.78)
2000-2004	-0.05(-0.05 to -0.05)	0.95(0.95 to 0.95)	-0.06(-0.07 to -0.06)	0.94(0.94 to 0.94)	-0.04(-0.04 to -0.03)	0.96(0.96 to 0.97)
2005-2009	0.13(0.12 to 0.13)	1.14(1.13 to 1.14)	0.14(0.13 to 0.14)	1.15(1.14 to 1.15)	0.12(0.11 to 0.12)	1.12(1.12 to 1.13)
2010-2014	0.26(0.25 to 0.26)	1.30(1.29 to 1.3)	0.28(0.27 to 0.29)	1.33(1.32 to 1.33)	0.23(0.23 to 0.24)	1.26(1.25 to 1.27)
2015-2019	0.43(0.42 to 0.44)	1.54(1.53 to 1.55)	0.45(0.44 to 0.47)	1.58(1.56 to 1.59)	0.4(0.39 to 0.41)	1.49(1.48 to 1.51)
Cohort						
1895-1899	1.75(1.7 to 1.81)	5.78(5.49 to 6.08)	1.79(1.69 to 1.89)	5.98(5.4 to 6.61)	1.72(1.66 to 1.78)	5.59(5.26 to 5.95)
1900-1994	1.68(1.65 to 1.71)	5.35(5.21 to 5.51)	1.73(1.69 to 1.78)	5.66(5.4 to 5.93)	1.65(1.61 to 1.69)	5.2(5.01 to 5.39)
1905-1909	1.56(1.54 to 1.58)	4.74(4.65 to 4.84)	1.61(1.58 to 1.64)	5.01(4.85 to 5.17)	1.53(1.5 to 1.56)	4.63(4.5 to 4.77)
1910-1914	1.44(1.42 to 1.45)	4.21(4.14 to 4.28)	1.47(1.45 to 1.5)	4.36(4.25 to 4.48)	1.43(1.4 to 1.45)	4.18(4.08 to 4.28)
1915-1919	1.29(1.28 to 1.31)	3.64(3.58 to 3.7)	1.32(1.29 to 1.34)	3.73(3.65 to 3.82)	1.3(1.28 to 1.32)	3.66(3.58 to 3.74)
1920-1924	1.16(1.15 to 1.17)	3.19(3.15 to 3.24)	1.19(1.17 to 1.21)	3.3(3.23 to 3.37)	1.16(1.14 to 1.18)	3.2(3.13 to 3.26)
1925-1929	0.97(0.96 to 0.99)	2.65(2.61 to 2.68)	1.01(0.99 to 1.03)	2.75(2.7 to 2.8)	0.97(0.95 to 0.98)	2.63(2.58 to 2.68)
1930-1934	0.78(0.77 to 0.8)	2.18(2.16 to 2.21)	0.81(0.79 to 0.83)	2.25(2.21 to 2.3)	0.76(0.74 to 0.78)	2.13(2.09 to 2.18)
1935-1939	0.59(0.58 to 0.61)	1.81(1.79 to 1.84)	0.62(0.6 to 0.64)	1.86(1.82 to 1.9)	0.56(0.54 to 0.58)	1.75(1.72 to 1.79)
1940-1944	0.44(0.42 to 0.45)	1.55(1.52 to 1.57)	0.46(0.44 to 0.48)	1.58(1.55 to 1.62)	0.40(0.38 to 0.43)	1.5(1.46 to 1.53)
1945-1949	0.3(0.28 to 0.31)	1.34(1.32 to 1.37)	0.31(0.28 to 0.33)	1.36(1.33 to 1.39)	0.26(0.24 to 0.29)	1.3(1.27 to 1.33)
1950-1954	0.12(0.1 to 0.14)	1.13(1.1 to 1.15)	0.13(0.1 to 0.15)	1.14(1.11 to 1.17)	0.09(0.06 to 0.12)	1.1(1.07 to 1.13)
1955-1959	-0.05(-0.07 to -0.03)	0.95(0.93 to 0.97)	-0.04(-0.07 to -0.01)	0.96(0.93 to 0.99)	-0.07(-0.1 to -0.04)	0.93(0.9 to 0.96)
1960-1964	-0.22(-0.25 to -0.2)	0.8(0.78 to 0.82)	-0.22(-0.26 to -0.19)	0.8(0.77 to 0.83)	-0.24(-0.27 to -0.2)	0.79(0.76 to 0.82)
1965-1969	-0.4(-0.43 to -0.37)	0.67(0.65 to 0.69)	-0.41(-0.44 to -0.37)	0.67(0.64 to 0.69)	-0.41(-0.45 to -0.36)	0.67(0.64 to 0.69)
1970-1974	-0.58(-0.61 to -0.55)	0.56(0.54 to 0.58)	-0.6(-0.64 to -0.56)	0.55(0.53 to 0.57)	-0.56(-0.61 to -0.52)	0.57(0.54 to 0.6)
1975-1979	-0.72(-0.76 to -0.69)	0.48(0.47 to 0.5)	-0.74(-0.79 to -0.7)	0.47(0.45 to 0.5)	-0.71(-0.76 to -0.66)	0.49(0.47 to 0.51)
1980-1984	-0.86(-0.9 to -0.82)	0.42(0.41 to 0.44)	-0.88(-0.93 to -0.83)	0.41(0.39 to 0.44)	-0.85(-0.91 to -0.8)	0.43(0.4 to 0.45)
1985-1989	-1.02(-1.07 to -0.98)	0.36(0.34 to 0.37)	-1.05(-1.11 to -1)	0.35(0.33 to 0.37)	-1.01(-1.07 to -0.94)	0.37(0.34 to 0.39)
1990-1994	-1.21(-1.26 to -1.16)	0.3(0.28 to 0.31)	-1.24(-1.31 to -1.17)	0.29(0.27 to 0.31)	-1.2(-1.28 to -1.12)	0.3(0.28 to 0.33)
1995-1999	-1.43(-1.5 to -1.36)	0.24(0.22 to 0.26)	-1.47(-1.57 to -1.37)	0.23(0.21 to 0.25)	-1.4(-1.51 to -1.29)	0.25(0.22 to 0.28)
2000-2004	-1.62(-1.73 to -1.51)	0.2(0.18 to 0.22)	-1.67(-1.81 to -1.52)	0.19(0.16 to 0.22)	-1.57(-1.73 to -1.4)	0.21(0.18 to 0.25)
2005-2009	-1.82(-2 to -1.64)	0.16(0.13 to 0.19)	-1.9(-2.15 to -1.65)	0.15(0.12 to 0.19)	-1.76(-2.02 to -1.5)	0.17(0.13 to 0.22)
2010-2014	-2.14(-2.44 to -1.85)	0.12(0.09 to 0.16)	-2.23(-2.62 to -1.84)	0.11(0.07 to 0.16)	-2.07(-2.5 to -1.63)	0.13(0.08 to 0.2)
AIC	17.86		12,99		15.21	
BIC	417.95		-55.89		197.96	
Deviance	740.01		266.17		520.02	

**Table 4:** CRC relative death risk due to age, period, and cohort effects.

Variables	Both		Male		Female	
	Coef_95%CI	RR_95%CI	Coef_95%CI	RR_95%CI	Coef_95%CI	RR_95%CI
Age						
5-9	-3.77(-3.95 to -3.6)	0.02(0.02 to 0.03)	-3.8(-4.04 to -3.57)	0.02(0.02 to 0.03)	-3.78(-4.04 to -3.51)	0.02(0.02 to 0.03)
10-14	-3.93(-4.09 to -3.78)	0.02(0.02 to 0.02)	-4.07(-4.29 to -3.85)	0.02(0.01 to 0.02)	-3.84(-4.07 to -3.61)	0.02(0.02 to 0.03)
15-19	-3.05(-3.15 to -2.96)	0.05(0.04 to 0.05)	-3.06(-3.18 to -2.93)	0.05(0.04 to 0.05)	-3.06(-3.2 to -2.91)	0.05(0.04 to 0.05)
20-24	-2.4(-2.47 to -2.34)	0.09(0.08 to 0.1)	-2.44(-2.54 to -2.35)	0.09(0.08 to 0.1)	-2.38(-2.48 to -2.27)	0.09(0.08 to 0.1)
25-29	-1.83(-1.89 to -1.78)	0.16(0.15 to 0.17)	-1.89(-1.96 to -1.82)	0.15(0.14 to 0.16)	-1.78(-1.86 to -1.7)	0.17(0.16 to 0.18)
30-34	-1.21(-1.25 to -1.17)	0.3(0.29 to 0.31)	-1.26(-1.32 to -1.2)	0.28(0.27 to 0.3)	-1.17(-1.23 to -1.1)	0.31(0.29 to 0.33)
35-39	-0.75(-0.78 to -0.71)	0.47(0.46 to 0.49)	-0.81(-0.86 to -0.76)	0.45(0.42 to 0.47)	-0.69(-0.75 to -0.64)	0.50(0.47 to 0.53)
40-44	-0.28(-0.32 to -0.25)	0.76(0.73 to 0.78)	-0.34(-0.38 to -0.3)	0.71(0.68 to 0.74)	-0.24(-0.28 to -0.19)	0.79(0.75 to 0.83)
45-49	0.2(0.17 to 0.23)	1.22(1.19 to 1.26)	0.16(0.12 to 0.2)	1.17(1.13 to 1.22)	0.23(0.19 to 0.27)	1.26(1.21 to 1.31)
50-54	0.62(0.6 to 0.64)	1.86(1.82 to 1.9)	0.62(0.59 to 0.64)	1.85(1.8 to 1.91)	0.61(0.58 to 0.64)	1.84(1.78 to 1.9)
55-59	0.99(0.97 to 1.01)	2.69(2.64 to 2.75)	1.03(1.01 to 1.05)	2.8(2.74 to 2.87)	0.92(0.89 to 0.95)	2.51(2.44 to 2.57)
60-64	1.29(1.28 to 1.31)	3.63(3.6 to 3.71)	1.37(1.35 to 1.39)	3.95(3.87 to 4.03)	1.17(1.15 to 1.19)	3.22(3.15 to 3.3)
65-69	1.52(1.51 to 1.53)	4.57(4.53 to 4.62)	1.63(1.61 to 1.64)	5.08(4.99 to 5.18)	1.39(1.37 to 1.41)	4.00(3.92 to 4.08)
70-74	1.71(1.7 to 1.73)	5.53(5.47 to 5.64)	1.82(1.8 to 1.84)	6.15(6.04 to 6.27)	1.6(1.58 to 1.62)	4.96(4.86 to 5.07)
75-79	1.91(1.89 to 1.92)	6.75(6.62 to 6.82)	2(1.98 to 2.03)	7.41(7.24 to 7.58)	1.84(1.81 to 1.86)	6.28(6.13 to 6.43)
80-84	2.06(2.04 to 2.08)	7.85(7.69 to 8)	2.14(2.11 to 2.16)	8.47(8.24 to 8.71)	2.03(2 to 2.06)	7.65(7.42 to 7.87)
85-89	2.2(2.18 to 2.23)	9.03(8.85 to 9.3)	2.25(2.22 to 2.29)	9.51(9.19 to 9.83)	2.22(2.19 to 2.26)	9.23(8.9 to 9.56)
90-94	2.32(2.29 to 2.35)	10.18(9.87 to 10.49)	2.3(2.26 to 2.34)	9.97(9.58 to 10.38)	2.39(2.35 to 2.44)	10.95(10.49 to 11.42)
95 plus	2.42(2.38 to 2.45)	11.25(10.8 to 11.59)	2.36(2.31 to 2.41)	10.56(10.04 to 11.1)	2.53(2.48 to 2.58)	12.52(11.9 to 13.18)
Period						
1990-1994	-0.37(-0.38 to -0.36)	0.69(0.68 to 0.7)	-0.4(-0.42 to -0.38)	0.67(0.66 to 0.68)	-0.34(-0.36 to -0.32)	0.71(0.7 to 0.73)
1995-1999	-0.21(-0.22 to -0.2)	0.81(0.80 to 0.82)	-0.22(-0.24 to -0.21)	0.8(0.79 to 0.81)	-0.2(-0.21 to -0.19)	0.82(0.81 to 0.83)
2000-2004	-0.04(-0.04 to -0.03)	0.96(0.96 to 0.97)	-0.05(-0.06 to -0.04)	0.95(0.95 to 0.96)	-0.03(-0.03 to -0.02)	0.98(0.97 to 0.98)
2005-2009	0.09(0.09 to 0.09)	1.09(1.09 to 1.09)	0.1(0.09 to 0.1)	1.10(1.09 to 1.11)	0.08(0.08 to 0.09)	1.09(1.08 to 1.09)
2010-2014	0.19(0.18 to 0.2)	1.21(1.2 to 1.22)	0.21(0.2 to 0.22)	1.23(1.22 to 1.25)	0.16(0.15 to 0.18)	1.18(1.16 to 1.19)
2015-2019	0.34(0.33 to 0.36)	1.40(1.39 to 1.43)	0.37(0.35 to 0.39)	1.44(1.42 to 1.47)	0.32(0.3 to 0.34)	1.37(1.35 to 1.4)
Cohort						
1895-1899	1.85(1.8 to 1.91)	6.36(6.05 to 6.75)	1.89(1.79 to 1.99)	6.63(6 to 7.34)	1.82(1.75 to 1.9)	6.2(5.75 to 6.68)
1900-1994	1.77(1.73 to 1.81)	5.87(5.64 to 6.11)	1.82(1.76 to 1.88)	6.18(5.83 to 6.56)	1.75(1.69 to 1.8)	5.74(5.43 to 6.08)
1905-1909	1.67(1.64 to 1.71)	5.31(5.16 to 5.53)	1.72(1.67 to 1.77)	5.59(5.34 to 5.85)	1.66(1.61 to 1.71)	5.25(5 to 5.51)
1910-1914	1.57(1.54 to 1.6)	4.81(4.66 to 4.95)	1.6(1.56 to 1.64)	4.96(4.76 to 5.16)	1.57(1.53 to 1.61)	4.81(4.6 to 5.02)
1915-1919	1.43(1.41 to 1.46)	4.18(4.1 to 4.31)	1.45(1.42 to 1.49)	4.28(4.13 to 4.44)	1.45(1.41 to 1.49)	4.25(4.08 to 4.42)
1920-1924	1.3(1.27 to 1.32)	3.67(3.56 to 3.74)	1.32(1.29 to 1.36)	3.75(3.63 to 3.88)	1.31(1.27 to 1.35)	3.7(3.56 to 3.84)
1925-1929	1.13(1.11 to 1.16)	3.1(3.03 to 3.19)	1.16(1.13 to 1.19)	3.18(3.08 to 3.29)	1.14(1.1 to 1.17)	3.12(3 to 3.23)
1930-1934	0.95(0.92 to 0.97)	2.59(2.51 to 2.64)	0.97(0.93 to 1)	2.63(2.54 to 2.72)	0.94(0.9 to 0.98)	2.56(2.46 to 2.65)
1935-1939	0.75(0.72 to 0.77)	2.12(2.05 to 2.16)	0.76(0.72 to 0.79)	2.14(2.06 to 2.21)	0.73(0.69 to 0.77)	2.07(1.99 to 2.15)
1940-1944	0.57(0.54 to 0.6)	1.77(1.72 to 1.82)	0.58(0.55 to 0.62)	1.79(1.73 to 1.86)	0.55(0.51 to 0.59)	1.73(1.66 to 1.81)
1945-1949	0.38(0.35 to 0.41)	1.46(1.42 to 1.51)	0.38(0.34 to 0.43)	1.47(1.41 to 1.53)	0.35(0.3 to 0.4)	1.42(1.35 to 1.49)
1950-1954	0.21(0.17 to 0.25)	1.23(1.19 to 1.28)	0.21(0.17 to 0.26)	1.24(1.18 to 1.3)	0.19(0.13 to 0.24)	1.20(1.14 to 1.27)
1955-1959	0.03(-0.01 to 0.07)	1.03(0.99 to 1.07)	0.03(-0.02 to 0.08)	1.03(0.98 to 1.09)	0(-0.06 to 0.06)	1(0.94 to 1.06)
1960-1964	-0.19(-0.23 to -0.15)	0.83(0.79 to 0.86)	-0.19(-0.25 to -0.13)	0.82(0.78 to 0.87)	-0.21(-0.28 to -0.14)	0.81(0.76 to 0.87)
1965-1969	-0.42(-0.47 to -0.37)	0.66(0.63 to 0.69)	-0.43(-0.5 to -0.36)	0.65(0.61 to 0.69)	-0.43(-0.51 to -0.36)	0.65(0.6 to 0.7)
1970-1974	-0.63(-0.69 to -0.58)	0.53(0.5 to 0.56)	-0.65(-0.73 to -0.58)	0.52(0.48 to 0.56)	-0.63(-0.71 to -0.55)	0.53(0.49 to 0.58)
1975-1979	-0.8(-0.86 to -0.74)	0.45(0.42 to 0.48)	-0.82(-0.9 to -0.74)	0.44(0.41 to 0.48)	-0.8(-0.89 to -0.71)	0.45(0.41 to 0.49)
1980-1984	-0.96(-1.03 to -0.89)	0.38(0.36 to 0.41)	-0.98(-1.07 to -0.89)	0.38(0.34 to 0.41)	-0.96(-1.06 to -0.86)	0.38(0.35 to 0.42)
1985-1989	-1.15(-1.23 to -1.07)	0.32(0.29 to 0.34)	-1.18(-1.28 to -1.08)	0.31(0.28 to 0.34)	-1.15(-1.26 to -1.03)	0.32(0.28 to 0.36)
1990-1994	-1.37(-1.47 to -1.28)	0.25(0.23 to 0.28)	-1.4(-1.53 to -1.27)	0.25(0.22 to 0.28)	-1.37(-1.52 to -1.23)	0.25(0.22 to 0.29)
1995-1999	-1.62(-1.76 to -1.48)	0.2(0.17 to 0.23)	-1.66(-1.85 to -1.48)	0.19(0.16 to 0.23)	-1.6(-1.81 to -1.39)	0.2(0.16 to 0.25)
2000-2004	-1.84(-2.05 to -1.63)	0.16(0.13 to 0.2)	-1.89(-2.16 to -1.61)	0.15(0.11 to 0.2)	-1.78(-2.09 to -1.47)	0.17(0.12 to 0.23)
2005-2009	-2.1(-2.44 to -1.77)	0.12(0.09 to 0.17)	-2.17(-2.62 to -1.71)	0.11(0.07 to 0.18)	-2.02(-2.5 to -1.53)	0.13(0.08 to 0.22)
2010-2014	-2.52(-3.08 to -1.95)	0.08(0.05 to 0.14)	-2.54(-3.29 to -1.8)	0.08(0.04 to 0.17)	-2.49(-3.37 to -1.62)	0.08(0.03 to 0.2)
AIC	13.01		10.94		11.46	
BIC	-48.52		-205.86		-141.95	
Deviance	273.54		116.20		180.11	

#### Period effect

In the period effects, both relative morbidity and mortality risks for CRC in Europe showed a gradual increase over time (**Figures 2B**, **3B**). The pattern of period effects was similar for relative morbidity and mortality risks. Relative morbidity risk increased from 0.61 to 1.54 and relative mortality risk increased from 0.69 to 1.40. 2005-2009, 2010-2014, and 2015-2019 were the three risk groups in which the relative morbidity and mortality risks were >1 for both men and women during this period.

#### Cohort effect

**Figures 2C** and **3C** show birth cohorts for relative incidence and mortality risks for each sex, respectively. Overall, the cohort effects all showed a consistent downward trend from 1895 to 2004. The risk of CRC incidence decreased from 5.78 in 1895-1899 to 0.48 in 2000-2004, and the risk of death decreased from 6.36 in 1895-1899 to 0.08 in 2000-2004. The 1895-1954 birth cohort was a risk group with an RR of incidence and death >1 in both sexes.

### Country-wise CRC burden in 2019

The epidemiology of CRC in different European countries is presented in the **Supplementary Material Table**. We found that the CRC burden varied considerably between countries. In terms of absolute counts, Germany was the leading country in 2019 with 78,951 (95% UI: 62,925 to 101,417) incident cases, followed by the Russian Federation (71,542 (62,884 to 81,644)) and Italy (60,514 (50,073 to 71,460)). San Marino (32 (95% UI: 25 to 42)), Monaco (57 (46 to 68)), and Andorra (79 (60 to 101)) had the lowest incident cases. The Russian Federation (42,834 (95% UI: 37,637 to 48,395)) was the leading country in terms of deaths, followed by Germany (37,552 (34,131 to 40,326)) and France (25,497 (22,330 to 27,996)). The three countries with the lowest number of deaths were San Marino (15 (11 to 21)), Monaco (25 (21 to 30)) and Andorra (34 (26 to 42)). The highest number of DALYs was recorded in the Russian Federation (939,798 (822,798 to 1,069,392)) followed by Germany (647,921 (598,987 to 694,996)) and Italy (436,750 (402,100 to 459,967)).

As for ASRs, in 2019, Monaco (60.69 (95% UI: 48.55 to 73.57)/100,000), Andorra (56.65 (42.79 to 71.90)/100,000) and Slovakia (56.45 (44.36 to 71.04)/100,000) had the highest ASIRs, while the countries with the lowest ASIRs were Albania (15.15 (11.4 to 19.9)/100,000), Republic of Moldova (29.17 (25.39 to 33.9)/100,000), and Slovakia (29.17 to 33.9 /100,000). Countries with the highest DALY rates were Hungary (630.26 (95% UI: 519.2 to 763.47)/100,000), followed by Bulgaria (582.28 (462.42 to 724.90)/100,000) and Slovakia (571.63 (449.13 to 723.92)/100,000). In addition, Hungary (28.56 (95% UI: 23.65 to 34.03)/100,000) reported the highest ASMR, followed by Slovakia (26.31 (20.96 to 32.80))/100,000) and Serbia (25.38 (20.63 to 31.01)/100,000). Albania (9.15 (95% UI: 6.96 to 11.89)/100,000), Iceland (11.81 (10.53 to 13.16)/100,000) and Switzerland (11.85 (10.7 to 12.84)/100,000) were the countries with the lowest ASMR.

### Country-wise temporal patterns of ASRs

From 1990 to 2019, ASIR increased in some countries. Notably, Romanian ASIR (AAPC: 2.81% (95% CI: 2.30% to 3.32%), *P* < 0.05), ASMR (1.77% (1.30% to 2.25%), *P* < 0.05) and age-standardized DALY rate (1.51% (1.11% to 1.91%), *P* < 0.05) increased most significantly. In addition, Cyprus and Bosnia and Herzegovina were among the top 3 countries in terms of ASIR increase. In contrast, Austria (AAPC: -1.56% (-1.97% to -1.14%), *P* < 0.05), Czechia (-0.70% (-1.31% to -0.09%), *P* < 0.05) and Luxembourg (-0.32% (-0.49% to -0.15%), *P* < 0.05) had the largest decline. Bosnia and Herzegovina and North Macedonia experienced the highest increase in both ASMR and age-standardized DALY rates. In contrast, Austria showed the largest decrease in ASMR (AAPC: -2.38% (-2.61% to -2.16%), *P* < 0.05) and age-standardized DALY rate (-2.59% (-2.84% to -2.35%), *P* < 0.05).

## Discussion

In this study, we analyzed trends in CRC burden in 44 European countries over the past three decades with the latest data from GBD 2019. At the same time, we revealed the epidemiological features of CRC in Europe by analyzing the age, period, and cohort effects of CRC morbidity and mortality. The results of the study can be used by local governments to target appropriate CRC prevention measures.

We found a significant increase in ASIR for CRC in Europe in the periods 1990-1994 and 1997-2003. ASIR appeared to stabilize after 2003, which was attributed to the rapid spread of CRC screening (19, 20). Nevertheless, ASIR for CRC in Europe was significantly higher than the worldwide average (38.38 *vs*. 26.71 per 100,000) (21), which may be related to the aging population, poor dietary habits, and lifestyle (22). Diets in European countries are characterized by high-calorie, high-fat, and high-protein diets that are too low in fiber, grains, fruits, and vegetables, which may be the main reason for the higher incidence of CRC in Europe (23, 24). There is growing evidence that fiber can alter the microbiota in the colon, thereby reducing the risk of CRC (25). Moreover, other risk factors such as obesity, physical inactivity, and smoking also increase the incidence of CRC (26, 27). During the study period, ASMR showed a decreasing trend, which is attributed to regular screening and improving diagnostic and therapeutic techniques (28).

Results of the age effect analysis showed an overall increasing trend in CRC incidence risk with increasing age in the European population. Higher age groups tend to have a combination of chronic underlying diseases, such as type 2 diabetes and hypertension, which, together with low levels of immune function, result in a higher incidence of CRC (29). Additionally, this study found that relative incidence risk was greater than 1 for both women over 50 years and men over 45 years, indicating that their risk of CRC incidence was higher than the overall average. There is no doubt that the middle-aged and older population will remain a priority population for CRC prevention and treatment in Europe in the future. Most guidelines recommend discontinuing cancer screening at age 75 years (30). The European Council recommended screening until the age of 75, and this cut-off is observed in most European countries (31). Given the life expectancy of the elderly, the risk of overdiagnosis is certainly greater. Most of the benefit-risk data for CRC screening over 75 years came from simulation studies (32). In our study, it found that age of 75 is the peak of CRC onset, which is correlated to the current status of CRC screening in the elderly. The surge in the relative incidence risk in the 95-plus age group is considered to be related to model prediction, and more large-sample cohort validation is needed in the future. It is noteworthy that in recent years a growing number of studies have shown an increasing incidence of CRC in young Europeans (33). It is recommended to strengthen health knowledge education for European residents, especially young adult males, to encourage them to develop good eating habits and lifestyles and maintain a healthy weight. It is also advisable to further increase CRC screening in the middle-aged and elderly population, to achieve early diagnosis and treatment.

Analysis of the period effect showed that the risk of CRC incidence and mortality in both men and women in Europe increased gradually over time. On the one hand, with the improvement of living standards, the dietary structure of residents has gradually changed, with a significant increase in the intake of fat, oil, salt, sugar, and red meat, and a decrease in the intake of fiber-rich foods such as vegetables and fruits. On the other hand, the rising work and mental stress accompanying economic development have also resulted in a marked increase in the incidence of poor lifestyles, such as alcohol intake, smoking, prolonged sedentary time, and staying up all night, which is a significant factor contributing to the increase in CRC incidence (34). A European Prospective Study on Cancer and Nutrition showed that increasing alcohol intake in middle and late adulthood increased CRC risk, whereas decreasing alcohol intake decreased the risk (35). Furthermore, the growing healthy life expectancy of the European population, the greater population aging, the expansion of CRC screening coverage, and the improvement of diagnostic techniques have also contributed to an increase in the number of reported cases, thus causing an increasing CRC incidence rate (19, 36).

The cohort effects analysis revealed a lower risk of CRC morbidity and mortality in the later birth cohort. Both men and women born in 2000-2004 had significantly lower risks of CRC morbidity and mortality compared to those born in 1895-1899. Earlier birth cohorts tend to be less well nourished than later birth cohorts due to factors such as war and famine, and nutritional status correlates with CRC risk. Studies have demonstrated that women who have experienced severe famine have a significantly higher risk of developing CRC than those who have not (37). Moreover, earlier-born populations are less literate, have a relatively poor awareness of CRC prevention, and are more likely to be exposed to CRC-related risk factors (38).

Previous studies have documented that men have a significantly higher incidence of CRC than women (1, 39). The present study also found that cases and rates of morbidity, mortality, and DALY were higher in European men than in women between 1990 and 2019. Bad habits such as smoking and alcohol consumption, which may contribute to the formation of CRC, are highly prevalent in the male population compared to females (26). Besides, sex hormones have been recognized as a factor in gender differences in CRC incidence and mortality (40). It has been shown that androgens negatively regulate the BMP signaling pathway by targeting the androgen receptor in intestinal stromal cells to promote proliferation and inhibit differentiation of intestinal stem cells, which provides a possible explanation for the high incidence of CRC in men (41). Another study found that a sex-biased gut microbiome may be a potential cause of sexual dimorphism in the development of CRC (42). Male mice showed significant enrichment of oncogenic bacteria and depletion of probiotics, which led to elevated levels of oncogenic lysophosphatidylcholine, promotion of cell proliferation, and impairment of intestinal barrier function, ultimately accelerating CRC tumorigenesis and increasing its mortality. Given these findings, it would be advisable to advocate for gender-specific public health campaigns or clinical guidelines.

Additionally, we found that the CRC burden varied by country. In 2019, Germany (78,951) had the highest incident cases. A population-based clinical cancer registry survey in Germany showed that, like many countries, socioeconomic inequalities in CRC survival exist in Germany (43). Further research into the underlying causes to overcome these inequalities is crucial. Hungary’s ASIR ranked 5th among 44 European countries, and Hungary reported the highest age-standardized DALY rate and ASMR. Hungary should promote the implementation of primary and secondary prevention and further expand the coverage of CRC screening to reduce the risk of death (44). ASIR and ASMR have stabilized or declined in most European countries, which may be attributable to the long-term implementation of colonoscopy and fecal examination screening programs. Notably, over the past 30 years, Romania has experienced the greatest increase in ASIR, ASMR, and age-standardized DALY rates. This upswing could be attributed to multiple factors such as the aging population, shifts in dietary habits, increased sedentary lifestyles, or the improved diagnostic facilities leading to heightened reported cases. Countries showcasing rising AAPC values in terms of incidence and DALY should take these as cues to be more proactive in reevaluating and reinforcing their healthcare strategies to better tackle this increasing burden. Conversely, Austria had the largest decrease in the above 3 indicators. CRC has been a serious public health problem in Romania, and it tops the list of gastrointestinal tract cancer deaths (45). Romania is estimated to have one of the highest CRC incidence and mortality rates in Europe, with a high mortality rate that is almost twice as high as the European range (46). Lifestyle factors, inadequate screening programs, and variations in treatment may account for the high incidence and mortality from CRC. The study noted that lifestyle changes such as tobacco and alcohol consumption, obesity and diabetes, sedentary lifestyles, and unhealthy dietary patterns may increase the prevalence and mortality of CRC (47). The north-central region of Romania is very developed and westernized, and the dietary intake of margarine, sausages, red meat, and a high-fat diet is associated with the incidence of CRC (48). Reducing alcohol consumption, maintaining good dietary habits and good weight management will make an important contribution to the prevention of CRC in Romania (49). It is also necessary to increase screening for the disease in young people and to raise the attention of clinicians to the increased incidence of CRC in young patients (50). In addition, attention has been paid to the phenomenon of genetic variability specific to the Romanian population, with studies on KRAS, NRAS, BRAF, PIK3CA, and TP53 Mutations (51). However, the exact mechanism remains unclear and further in-depth studies on the causes of the high incidence of CRC in Romania are highly relevant.

In conclusion, although the incidence of CRC in Europe seems to have generally stabilized in recent years, it still faces a severe disease burden, and there is still a long way to go in CRC prevention and control, which requires the development of targeted prevention and treatment strategies based on gender and age. Prevention and early diagnosis of CRC in European populations, especially in middle-aged and elderly men, should continue to be strengthened. Currently, secondary prevention based on population-based screening is still the main preventive measure for CRC and the most powerful measure to reduce CRC morbidity and mortality. Continuing to refine the screening and management of patients with high CRC will facilitate further reduction of CRC burden.

## Limitation

There are some limitations of this study. First, the quality of the data used in this study relies on the quality control of the original GBD data collection process, and the bias is still inevitable. It is recommended that the findings of this study be further validated with the help of a large cohort study. Secondly, this study only focused on the current situation of the European population. In the future, we need to use more types of data to build relevant models to predict the prevalence of CRC and provide a richer basis for CRC prevention and control. Finally, due to the lack of data, we were unable to determine CRC subtype burden by histological classification of tumors or specific anatomical sites.

## Conclusion

ASIR for CRC in Europe generally trended upwards from 1990 to 2019, stabilizing in recent years but still at a high level. Absolute counts and ASRs of CRC varied considerably in different European countries. Incidence and mortality risks increased with age in both men and women. In terms of gender differences, the CRC burden was significantly higher in European men than in women. CRC disease burden in Europe was of concern and preventive and control measures should be taken according to its epidemiological features. Middle-aged and older men should be a priority population for the prevention and treatment of CRC in Europe.

## Data availability statement

The original contributions presented in the study are included in the article/**Supplementary Material**. Further inquiries can be directed to the corresponding authors.

## Author contributions

DL: Conceptualization, Writing – original draft. CM: Data curation, Writing – original draft. ZZ: Investigation. YL: Formal analysis. JL: Methodology. YX: Funding acquisition, Writing – review & editing. YZ: Funding acquisition, Writing – review & editing.

## References

[1] Sung H Ferlay J Siegel RL Laversanne M Soerjomataram I Jemal A. Global Cancer Statistics 2020: GLOBOCAN estimates of incidence and mortality worldwide for 36 cancers in 185 countries. CA Cancer J Clin (2021) 71(3):209–49. doi: 10.3322/caac.21660 33538338

[2] Chaudhuri D Sasaki K Karkar A Sharif S Lewis K Mammen MJ. Corticosteroids in COVID-19 and non-COVID-19 ARDS: a systematic review and meta-analysis. Intensive Care Med (2021) 47(5):521–37. doi: 10.1007/s00134-021-06394-2 PMC8054852 33876268

[3] Yu XQ Weber M Smith D Velentzis L Kliewer EV David M. Incidence profile of four major cancers among migrants in Australia, 2005-2014. J Cancer Res Clin Oncol (2023) 149(11):8317–25. doi: 10.1007/s00432-023-04764-5 PMC10374701 37072554

[4] Henderson RH French D Maughan T Adams R Allemani C Minicozzi P. The economic burden of colorectal cancer across Europe: a population-based cost-of-illness study. Lancet Gastroenterol Hepatol (2021) 6(9):709–22. doi: 10.1016/S2468-1253(21)00147-3 34329626

[5] Safiri S Carson-Chahhoud K Noori M Nejadghaderi SA Sullman M Ahmadian Heris J. Burden of chronic obstructive pulmonary disease and its attributable risk factors in 204 countries and territories, 1990-2019: results from the Global Burden of Disease Study 2019. BMJ (2022) 378:e069679. doi: 10.1136/bmj-2021-069679 35896191 PMC9326843

[6] Li Y Zheng J Deng Y Deng X Lou W Wei B. Global burden of female breast cancer: age-period-cohort analysis of incidence trends from 1990 to 2019 and forecasts for 2035. Front Oncol (2022) 12:891824. doi: 10.3389/fonc.2022.891824 35756641 PMC9218744

[7] Wu Y Deng Y Wei B Xiang D Hu J Zhao P. Global, regional, and national childhood cancer burden, 1990-2019: An analysis based on the Global Burden of Disease Study 2019. J Adv Res (2022) 40:233–47. doi: 10.1016/j.jare.2022.06.001 PMC9481947 35700919

[8] Heidari-Foroozan M Saeedi Moghaddam S Keykhaei M Shobeiri P Azadnajafabad S Esfahani Z. Regional and national burden of leukemia and its attributable burden to risk factors in 21 countries and territories of North Africa and Middle East, 1990-2019: results from the GBD study 2019. J Cancer Res Clin Oncol (2023) 149(8):4149–61. doi: 10.1007/s00432-022-04293-7 PMC11797763 36048271

[9] GBD 2019 Demographics Collaborators. Global age-sex-specific fertility, mortality, healthy life expectancy (HALE), and population estimates in 204 countries and territories, 1950-2019: a comprehensive demographic analysis for the Global Burden of Disease Study 2019. Lancet (2020) 396(10258):1160–203. doi: 10.1016/S0140-6736(20)30977-6 PMC7566045 33069325

[10] GBD 2019 Diseases and Injuries Collaborators. Global burden of 369 diseases and injuries in 204 countries and territories, 1990-2019: a systematic analysis for the Global Burden of Disease Study 2019. Lancet (2020) 396(10258):1204–22. doi: 10.1016/S0140-6736(20)30925-9 PMC7567026 33069326

[11] Kim HJ Fay MP Feuer EJ Midthune DN. Permutation tests for joinpoint regression with applications to cancer rates. Stat Med (2000) 19(3):335–51. doi: 10.1002/(sici)1097-0258(20000215)19:3&lt;335::aid-sim336<3.0.co;2-z 10649300

[12] Qiu H Cao S Xu R. Cancer incidence, mortality, and burden in China: a time-trend analysis and comparison with the United States and United Kingdom based on the global epidemiological data released in 2020. Cancer Commun (Lond) (2021) 41(10):1037–48. doi: 10.1002/cac2.12197 PMC8504144 34288593

[13] Mubarik S Yu Y Wang F Malik SS Liu X Fawad M. Epidemiological and sociodemographic transitions of female breast cancer incidence, death, case fatality and DALYs in 21 world regions and globally, from 1990 to 2017: An Age-Period-Cohort Analysis. J Adv Res (2022) 37:185–96. doi: 10.1016/j.jare.2021.07.012 PMC9039678 35499053

[14] Hu W Fang L Zhang H Ni R Pan G. Global disease burden of COPD from 1990 to 2019 and prediction of future disease burden trend in China. Public Health (2022) 208:89–97. doi: 10.1016/j.puhe.2022.04.015 35728417

[15] Zhang Y Liu J Han X Jiang H Zhang L Hu J. Long-term trends in the burden of inflammatory bowel disease in China over three decades: A joinpoint regression and age-period-cohort analysis based on GBD 2019. Front Public Health (2022) 10:994619. doi: 10.3389/fpubh.2022.994619 36159285 PMC9490087

[16] Dong Z Wang QQ Yu SC Huang F Liu JJ Yao HY. Age-period-cohort analysis of pulmonary tuberculosis reported incidence, China, 2006-2020. Infect Dis Poverty (2022) 11(1):85. doi: 10.1186/s40249-022-01009-4 35902982 PMC9331155

[17] Metcalfe A Ahmed SB Nerenberg K. Age-period-cohort effects in pre-existing and pregnancy-associated diseases amongst primiparous women. Biol Sex Differ (2020) 11(1):19. doi: 10.1186/s13293-020-00293-9 32307020 PMC7168828

[18] Li Y Ren N Zhang B Yang C Li A Li X. Gastric cancer incidence trends in China and Japan from 1990 to 2019: Disentangling age-period-cohort patterns. Cancer (2023) 129(1):98–106. doi: 10.1002/cncr.34511 36284481

[19] Altobelli E Lattanzi A Paduano R Varassi G di Orio F. Colorectal cancer prevention in Europe: burden of disease and status of screening programs. Prev Med (2014) 62:132–41. doi: 10.1016/j.ypmed.2014.02.010 24530610

[20] Kaminski MF Robertson DJ Senore C Rex DK. Optimizing the quality of colorectal cancer screening worldwide. Gastroenterology (2020) 158(2):404–17. doi: 10.1053/j.gastro.2019.11.026 31759062

[21] Colorectal Cancer Collaborators. Global, regional, and national burden of colorectal cancer and its risk factors, 1990-2019: a systematic analysis for the Global Burden of Disease Study 2019. Lancet Gastroenterol Hepatol (2022) 7(7):627–47. doi: 10.1016/S2468-1253(22)00044-9 PMC9192760 35397795

[22] Lee S Meyerhardt JA. Impact of diet and exercise on colorectal cancer. Hematol Oncol Clin North Am (2022) 36(3):471–89. doi: 10.1016/j.hoc.2022.02.004 35504785

[23] Patel SG Karlitz JJ Yen T Lieu CH Boland CR. The rising tide of early-onset colorectal cancer: a comprehensive review of epidemiology, clinical features, biology, risk factors, prevention, and early detection. Lancet Gastroenterol Hepatol (2022) 7(3):262–74. doi: 10.1016/S2468-1253(21)00426-X 35090605

[24] Wang L Lo CH He X Hang D Wang M Wu K. Risk factor profiles differ for cancers of different regions of the colorectum. Gastroenterology (2020) 159(1):241–56.e13. doi: 10.1053/j.gastro.2020.03.054 32247020 PMC7387153

[25] Makki K Deehan EC Walter J Bäckhed F. The impact of dietary fiber on gut microbiota in host health and disease. Cell Host Microbe (2018) 23(6):705–15. doi: 10.1016/j.chom.2018.05.012 29902436

[26] Dekker E Tanis PJ Vleugels J Kasi PM Wallace MB. Colorectal cancer. Lancet (2019) 394(10207):1467–80. doi: 10.1016/S0140-6736(19)32319-0 31631858

[27] Li H Boakye D Chen X Jansen L Chang-Claude J Hoffmeister M. Associations of body mass index at different ages with early-onset colorectal cancer. Gastroenterology (2022) 162(4):1088–97.e3. doi: 10.1053/j.gastro.2021.12.239 34914944

[28] Kanth P Inadomi JM. Screening and prevention of colorectal cancer. BMJ (2021) 374:n1855. doi: 10.1136/bmj.n1855 34526356

[29] O'Sullivan DE Sutherland RL Town S Chow K Fan J Forbes N. Risk factors for early-onset colorectal cancer: A systematic review and meta-analysis. Clin Gastroenterol Hepatol (2022) 20(6):1229–40.e5. doi: 10.1016/j.cgh.2021.01.037 33524598

[30] Ferlay J Colombet M Soerjomataram I Dyba T Randi G Bettio M. Cancer incidence and mortality patterns in Europe: Estimates for 40 countries and 25 major cancers in 2018. Eur J Cancer (2018) 103:356–87. doi: 10.1016/j.ejca.2018.07.005 30100160

[31] Altobelli E D'Aloisio F Angeletti PM. Colorectal cancer screening in countries of European Council outside of the EU-28. World J Gastroenterol (2016) 22(20):4946–57. doi: 10.3748/wjg.v22.i20.4946 PMC4873887 27239121

[32] Guittet L Quipourt V Aparicio T Carola E Seitz JF Paillaud E. Should we screen for colorectal cancer in people aged 75 and over? A systematic review - collaborative work of the French geriatric oncology society (SOFOG) and the French federation of digestive oncology (FFCD). BMC Cancer (2023) 23(1):17. doi: 10.1186/s12885-022-10418-5 36604640 PMC9817257

[33] Vuik FE Nieuwenburg SA Bardou M Lansdorp-Vogelaar I Dinis-Ribeiro M Bento MJ. Increasing incidence of colorectal cancer in young adults in Europe over the last 25 years. Gut (2019) 68(10):1820–6. doi: 10.1136/gutjnl-2018-317592 PMC6839794 31097539

[34] Yu J Feng Q Kim JH Zhu Y. Combined effect of healthy lifestyle factors and risks of colorectal adenoma, colorectal cancer, and colorectal cancer mortality: systematic review and meta-analysis. Front Oncol (2022) 12:827019. doi: 10.3389/fonc.2022.827019 35936678 PMC9353059

[35] Mayén AL Viallon V Botteri E Proust-Lima C Bagnardi V Batista V. A longitudinal evaluation of alcohol intake throughout adulthood and colorectal cancer risk. Eur J Epidemiol (2022) 37(9):915–29. doi: 10.1007/s10654-022-00900-6 36063305

[36] Cardoso R Guo F Heisser T Hackl M Ihle P De Schutter H. Colorectal cancer incidence, mortality, and stage distribution in European countries in the colorectal cancer screening era: an international population-based study. Lancet Oncol (2021) 22(7):1002–13. doi: 10.1016/S1470-2045(21)00199-6 34048685

[37] Brand MP Peeters PH van Gils CH Elias SG. Pre-adult famine exposure and subsequent colorectal cancer risk in women. Int J Epidemiol (2017) 46(2):612–21. doi: 10.1093/ije/dyw121 27585673

[38] Kirkegaard P Mortensen GL Mortensen SL Larsen MB Gabel P Andersen B. Making decisions about colorectal cancer screening. A qualitative study among citizens with lower educational attainment. Eur J Public Health (2016) 26(1):176–81. doi: 10.1093/eurpub/ckv207 26541860

[39] Chen X Heisser T Cardoso R Hoffmeister M Brenner H. Overall and age-specific risk advancement periods of colorectal cancer for men vs women: Implications for gender-sensitive screening offers. Int J Cancer (2023) 153(3):547–51. doi: 10.1002/ijc.34455 36727542

[40] Hang D Shen H. Sex hormone and colorectal cancer: the knowns and unknowns. Cancer Epidemiol Biomarkers Prev (2021) 30(7):1302–4. doi: 10.1158/1055-9965.EPI-21-0472 34210680

[41] Yu X Li S Xu Y Zhang Y Ma W Liang C. Androgen maintains intestinal homeostasis by inhibiting BMP signaling via intestinal stromal cells. Stem Cell Rep (2020) 15(4):912–25. doi: 10.1016/j.stemcr.2020.08.001 PMC7561494 32916121

[42] Wang L Tu YX Chen L Zhang Y Pan XL Yang SQ. Male-biased gut microbiome and metabolites aggravate colorectal cancer development. Adv Sci (Weinh) (2023) 10 (25):e2206238. doi: 10.1002/advs.202206238 37400423 PMC10477899

[43] Jansen L Behrens G Finke I Maier W Gerken M Pritzkuleit R. Area-based socioeconomic inequalities in colorectal cancer survival in Germany: investigation based on population-based clinical cancer registration. Front Oncol (2020) 10:857. doi: 10.3389/fonc.2020.00857 32670870 PMC7326086

[44] Csanádi M Gini A Koning H Széles G Pitter JG Oroszi B. Modeling costs and benefits of the organized colorectal cancer screening programme and its potential future improvements in Hungary. J Med Screen (2021) 28(3):268–76. doi: 10.1177/0969141320968598 33153369

[45] Vălean S Armean P Resteman S Nagy G Mureşan A Mircea PA. Cancer mortality in Romania, 1955-2004. Digestive sites: esophagus, stomach, colon and rectum, pancreas, liver, gallbladder and biliary tree. J Gastrointestin Liver Dis (2008) 17(1):9–14. 18392237

[46] Ionescu EM Tieranu CG Maftei D Grivei A Olteanu AO Arbanas T. Colorectal cancer trends of 2018 in Romania-an important geographical variation between Northern and Southern lands and high mortality versus European averages. J Gastrointest Cancer (2021) 52(1):222–8. doi: 10.1007/s12029-020-00382-3 32152824

[47] Song R Petimar J Wang M Tabung FK Song M Liu L. Adherence to the world cancer research fund/American institute for cancer research cancer prevention recommendations and colorectal cancer survival. Cancer Epidemiol Biomarkers Prev (2021) 30(10):1816–25. doi: 10.1158/1055-9965.EPI-21-0120 PMC8492523 34272268

[48] Fira-Mladinescu C Fira-Mladinescu O Doroftei S Sas F Ursoniu S Ionuţ R. [Food intake and colorectal cancers; an ecological study in Romania]. Rev Med Chir Soc Med Nat Iasi (2008) 112(3):805–11. 20201272

[49] Lotrean LM Florea M Lencu C. Lifestyle and cancer prevention-opinions and behaviors among Romanian university students. Int J Gen Med (2021) 14:1525–32. doi: 10.2147/IJGM.S303094 PMC8079252 33935514

[50] Gheju A Jurescu A Tăban S Al-Jobory D Lazăr F Dema A. Different disease characteristics in young patients with colorectal cancer: a large retrospective study in a city in Romania. J Int Med Res (2021) 49(5):3000605211016630. doi: 10.1177/03000605211016630 34034541 PMC8161876

[51] Afrăsânie VA Marinca MV Gafton B Alexa-Stratulat T Rusu A Froicu EM. Clinical, pathological and molecular insights on KRAS, NRAS, BRAF, PIK3CA and TP53 mutations in metastatic colorectal cancer patients from Northeastern Romania. Int J Mol Sci (2023) 24(16):12679. doi: 10.3390/ijms241612679 PMC10454287 37628868

