# Supplementary material for: Long-term trends in the burden of colorectal cancer in Europe over three decades: a joinpoint regression and age-period-cohort analysis
Source: Front Oncol. 2023 Dec 5;13:1287653. doi: 10.3389/fonc.2023.1287653 (PMC10728819; doi:10.3389/fonc.2023.1287653)
Supplement: Supplementary file 1 [file Table_1.docx]

**Supplementary Table 1** CRC incidence in 44 European countries in 1990 and 2019 and AAPC from 1990 to 2019

| Incidence |  |  |  |  |  |
| --- | --- | --- | --- | --- | --- |
| location | 1990 |  | 2019 |  | 1990-2019 |
|  | Incidence cases NO.(95%UI) | ASIR/100,000 (95% UI) | Incidence cases NO.(95%UI) | ASIR/100,000 (95% UI) | AAPC (95%CI) |
| Monaco | 31.84 (25.32 to 38.52) | 45.64 (36.26 to 55.36) | 56.94 (46.01 to 68.07) | 60.69 (48.55 to 73.57) | 1 (0.93 to 1.07) |
| Andorra | 28.63 (22.07 to 38.38) | 53.3 (41.79 to 70.73) | 79.46 (60.28 to 100.67) | 56.65 (42.79 to 71.9) | 0.21 (0.02 to 0.39) |
| Slovakia | 2282.1 (2171.14 to 2402.39) | 38.16 (36.33 to 40.06) | 5202.76 (4086.97 to 6574.31) | 56.45 (44.36 to 71.04) | 1.42 (1.08 to 1.76) |
| Netherlands | 8766.4 (8319.03 to 9148.97) | 43.93 (41.77 to 45.81) | 18773.93 (14703.4 to 23598.15) | 55.39 (43.29 to 69.76) | 0.72 (0.41 to 1.03) |
| Hungary | 6157.42 (5919.26 to 6404.89) | 42.22 (40.57 to 43.89) | 9872.57 (8137.25 to 11904.63) | 52.24 (42.96 to 62.9) | 0.62 (0.36 to 0.89) |
| Croatia | 2204.5 (2071.4 to 2346.46) | 35.03 (32.89 to 37.28) | 4409.03 (3503.08 to 5472.45) | 50.47 (39.91 to 62.58) | 1.58 (0.73 to 2.43) |
| Spain | 19094.47 (18176.05 to 19858.4) | 35.28 (33.51 to 36.64) | 47432.09 (37492.96 to 60136.9) | 50.14 (39.34 to 63.84) | 1.27 (0.9 to 1.65) |
| San Marino | 14.8 (12.84 to 16.94) | 44.35 (38.44 to 50.48) | 32.11 (24.87 to 42.07) | 49.55 (37.89 to 65.53) | 0.39 (0.23 to 0.55) |
| Norway | 2790.69 (2644.85 to 2905.49) | 40.85 (38.89 to 42.41) | 4745.54 (4025.82 to 5535.1) | 49.46 (41.9 to 57.73) | 0.59 (0.35 to 0.84) |
| Denmark | 3159.64 (3026.16 to 3282.87) | 39 (37.42 to 40.5) | 5576.78 (4363.77 to 7016.87) | 48.41 (37.76 to 61.15) | 0.63 (0.29 to 0.98) |
| Ireland | 1779.17 (1692.21 to 1861.33) | 43.76 (41.69 to 45.74) | 3431.44 (2687 to 4294.68) | 45.95 (35.89 to 57.72) | -0.01 (-0.19 to 0.17) |
| Portugal | 4364.72 (4178.56 to 4541.72) | 32 (30.61 to 33.25) | 10244.7 (8093.66 to 12945.77) | 45.29 (35.23 to 57.72) | 1.09 (0.95 to 1.24) |
| Bulgaria | 3518.58 (3329.17 to 3703.71) | 28.16 (26.65 to 29.6) | 6198.88 (4975.35 to 7640.55) | 43.84 (35.01 to 54.21) | 1.59  (1.28 to 1.9) |
| United Kingdom | 38717.63 (37267.8 to 39640.39) | 42.73 (41.17 to 43.7) | 54428.88 (45869.58 to 63942.15) | 43.6 (36.6 to 51.5) | 0.13 (-0.18 to 0.43) |
| Serbia | 3075.44 (2523.83 to 3443.37) | 27.33 (22.22 to 30.62) | 6788.95 (5452.18 to 8445.52) | 43.55 (35.07 to 54.26) | 1.66 (1.39 to 1.94) |
| Italy | 33947.06 (32569.17 to 35072.09) | 38.37 (36.84 to 39.6) | 60514.4 (50072.91 to 71460.43) | 43.54 (35.99 to 51.56) | 0.35 (0.23 to 0.47) |
| Estonia | 633.81 (603 to 663.52) | 30.9 (29.45 to 32.33) | 1117.51 (892.33 to 1390.52) | 42.21 (33.4 to 52.75) | 1.15 (0.49 to 1.81) |
| Czechia | 6723.43 (6471.54 to 6957.47) | 48.79 (46.99 to 50.49) | 8839.33 (7287.79 to 10730.15) | 42.14 (34.45 to 51.15) | -0.7 (-1.31 to -0.09) |
| Germany | 55726.36 (53109.79 to 57807.75) | 43.58 (41.64 to 45.17) | 78951.36 (62925.15 to 101416.8) | 41.4 (32.67 to 53.84) | -0.28 (-0.75 to 0.2) |
| Slovenia | 803.45 (620.57 to 1024.06) | 32.94 (25.35 to 41.98) | 1726.16 (1346.51 to 2221.52) | 41.05 (31.88 to 52.95) | 0.45 (0.07 to 0.83) |
| Belgium | 6564.32 (6198.04 to 6876.1) | 42.43 (40.15 to 44.39) | 8993.96 (7126.21 to 11223.44) | 39.33 (30.86 to 49.46) | -0.32 (-0.49 to -0.15) |
| France | 32302.05 (30505.88 to 33815.87) | 38.68 (36.69 to 40.42) | 52276.87 (40682.34 to 65961.76) | 38.47 (30.1 to 49.3) | -0.1 (-0.21 to 0.01) |
| Cyprus | 160.06 (141.01 to 181.97) | 20.45 (18.17 to 23.34) | 739.03 (637.73 to 843.46) | 38.15 (33.1 to 43.43) | 2.24 (1.75 to 2.73) |
| Luxembourg | 245.3 (228.91 to 261.87) | 44.89 (41.96 to 47.94) | 372.36 (302.41 to 450.71) | 37.17 (30.27 to 45.07) | -0.66 (-1.08 to -0.23) |
| Sweden | 5049.14 (4767.15 to 5279.93) | 33.51 (31.71 to 34.94) | 7720.11 (6589.95 to 8929.93) | 36.99 (31.41 to 42.78) | 0.27 (0.04 to 0.5) |
| Romania | 4862.44 (4676.49 to 5042.6) | 17.29 (16.62 to 17.92) | 13038.52 (10746.42 to 15541.48) | 36.16 (29.52 to 43.3) | 2.81 (2.3 to 3.32) |
| North Macedonia | 359.92 (328.66 to 387.5) | 19.27 (17.6 to 20.75) | 1134.79 (898.34 to 1416.17) | 35.36 (28.19 to 43.94) | 2.12 (1.93 to 2.3) |
| Poland | 10521.17 (10183.55 to 10780.06) | 24.23 (23.38 to 24.84) | 24276.57 (20748.27 to 28769.21) | 34.96 (29.84 to 41.55) | 1.2 (0.95 to 1.44) |
| Bosnia and Herzegovina | 722.56 (683.51 to 765.51) | 18.01 (17.01 to 19.13) | 2057.52 (1618.9 to 2570.85) | 34.91 (27.54 to 43.53) | 2.22  (1.87 to 2.57) |
| Belarus | 3387.04 (3252.73 to 3534.12) | 26.19 (25.17 to 27.28) | 5395.65 (4321.97 to 6836.97) | 34.12 (27.11 to 43.36) | 1.04 (0.4 to 1.7) |
| Israel | 1537.65 (1453.23 to 1618.8) | 31.87 (30.08 to 33.55) | 3893.19 (3050.35 to 4899.63) | 33.64 (26.3 to 42.59) | 0.06 (-0.31 to 0.44) |
| Switzerland | 3206.43 (3023.38 to 3380.55) | 30.71 (29.09 to 32.26) | 5823.85 (4528.8 to 7396.41) | 33.59 (25.91 to 42.71) | 0.21 (0.09 to 0.33) |
| Greece | 3842.91 (3639.65 to 4034.39) | 25.22 (23.89 to 26.45) | 7902.55 (6276.25 to 9939.99) | 33.21 (26.1 to 42.09) | 0.71 (0.56 to 0.87) |
| Austria | 5880.99 (5618.8 to 6151.36) | 50.14 (47.99 to 52.36) | 5847.01 (4783.48 to 7108.92) | 33.07 (26.71 to 40.55) | -1.56 (-1.97 to -1.14) |
| Malta | 119.53 (110.11 to 129.53) | 28.1 (25.83 to 30.43) | 303.34 (255.52 to 360.81) | 32.92 (27.83 to 39.12) | 0.58 (0.34 to 0.81) |
| Finland | 1867.7 (1782.17 to 1951.96) | 26.14 (24.95 to 27.3) | 3799.35 (2973.47 to 4804.78) | 31.46 (24.55 to 40) | 0.7 (0.64 to 0.77) |
| Ukraine | 21516.18 (20700.39 to 22345.36) | 30.04 (28.99 to 31.17) | 23387.64 (19879.5 to 27253.49) | 31.27 (26.56 to 36.54) | 0.19 (-0.45 to 0.83) |
| Russian Federation | 41738.59 (40275.83 to 43259.1) | 23 (22.17 to 23.86) | 71542.21 (62883.73 to 81644.42) | 30.77 (27.02 to 35.11) | 1.32 (0.34 to 2.32) |
| Iceland | 84.66 (78.19 to 92.18) | 29.62 (27.3 to 32.18) | 169.15 (146.69 to 195.25) | 30.71 (26.82 to 35.36) | 0.13 (-0.14 to 0.39) |
| Latvia | 905.16 (866.18 to 945.3) | 25.33 (24.25 to 26.45) | 1213.99 (1015.23 to 1460.84) | 30.54 (25.44 to 36.96) | 0.74 (-0.11 to 1.58) |
| Montenegro | 135.11 (113.52 to 154.61) | 21.79 (18.27 to 24.88) | 299.45 (245.01 to 361.95) | 30.47 (24.96 to 36.76) | 1.25 (0.93 to 1.58) |
| Lithuania | 1106.93 (1060.33 to 1156.48) | 24.65 (23.6 to 25.8) | 1668.88 (1384.75 to 2008.33) | 29.2 (24.01 to 35.37) | 0.67 (-0.1 to 1.44) |
| Republic of Moldova | 1113.08 (1067.53 to 1165.83) | 24.62 (23.62 to 25.77) | 1691.2 (1472.09 to 1927.05) | 29.17 (25.39 to 33.14) | 0.98 (-0.27 to 2.25) |
| Albania | 219.59 (204.3 to 234.94) | 10.53 (9.74 to 11.29) | 629.63 (470.32 to 827.83) | 15.15 (11.4 to 19.9) | 1.25 (0.73 to 1.77) |

**Supplementary Table 2** CRC DALY in 44 European countries in 1990 and 2019 and AAPC from 1990 to 2019

| DALY |  |  |  |  |  |
| --- | --- | --- | --- | --- | --- |
| location | 1990 |  | 2019 |  | 1990-2019 |
|  | DALY cases NO.(95%UI) | ASR/100,000 (95% UI) | DALY cases NO.(95%UI) | ASR/100,000 (95% UI) | AAPC (95%CI) |
| Hungary | 95738.16 (92440.17 to 99057.96) | 662.19 (639.43 to 684.89) | 115100.97 (94609.24 to 138287.29) | 630.26 (519.2 to 763.47) | -0.22 (-0.53 to 0.1) |
| Bulgaria | 59038.04 (56002.57 to 61950.19) | 476.62 (452.59 to 499.88) | 78043.16 (62257.42 to 96599.91) | 582.28 (462.42 to 724.9) | 0.74 (0.29 to 1.19) |
| Slovakia | 33243.14 (31759.56 to 34894.86) | 558.22 (533.57 to 585.41) | 51875.19 (40791.38 to 65885.31) | 571.63 (449.13 to 723.92) | 0.08 (-0.27 to 0.42) |
| Serbia | 56032.63 (46086.02 to 62853.27) | 488.71 (400.55 to 550.21) | 84595.85 (67489.7 to 105390.84) | 554.79 (441.74 to 693.84) | 0.39 (0.02 to 0.75) |
| Croatia | 31342.75 (29503.33 to 33327.22) | 492.13 (463.51 to 523.46) | 44361.72 (35210.61 to 55454.83) | 529.25 (415.54 to 665.79) | 0.51 (-0.23 to 1.25) |
| Poland | 208520.21 (203260.63 to 212561.48) | 477.99 (465.58 to 487.34) | 350819.3 (296650.44 to 412619.34) | 517.99 (437.26 to 610.63) | 0.28 (0.13 to 0.42) |
| Monaco | 307.8 (244.62 to 372.53) | 473.45 (377.85 to 572.06) | 435.61 (350.15 to 522.76) | 494.14 (392.6 to 604.35) | 0.15 (0.12 to 0.18) |
| Bosnia and Herzegovina | 14309.61 (13549.28 to 15122.98) | 335.07 (317.61 to 355.24) | 28248.89 (22240.89 to 35384.85) | 490.14 (386.13 to 613.75) | 1.26 (0.77 to 1.75) |
| North Macedonia | 6872.56 (6289.38 to 7399.92) | 354.5 (324.6 to 381.82) | 15695.43 (12332.68 to 19726.94) | 487.48 (386.3 to 610.72) | 1.1 (0.92 to 1.29) |
| Ukraine | 387420.49 (374495.66 to 400463.46) | 549.39 (531.06 to 568.2) | 350467.05 (300710.78 to 412416.93) | 485.29 (416.28 to 572.27) | -0.24 (-0.94 to 0.47) |
| Romania | 91439.59 (88161.83 to 94655.43) | 324.54 (313.35 to 335.44) | 163169.1 (133788.89 to 196692.9) | 474.35 (387.34 to 575.79) | 1.51 (1.11 to 1.91) |
| Andorra | 322.98 (248.09 to 433.65) | 581.93 (450.2 to 772.22) | 649.24 (496.53 to 829.15) | 466.28 (355.25 to 596.45) | -0.76 (-0.85 to -0.68) |
| Czechia | 101988.83 (98642.26 to 105224.49) | 749.25 (724.47 to 773.01) | 91539.53 (75040.1 to 111252.61) | 452.09 (370.11 to 551.18) | -2.01 (-2.51 to -1.51) |
| Republic of Moldova | 21631.62 (20779.61 to 22591.47) | 469.65 (452.04 to 489.52) | 24957.11 (21688.87 to 28418.98) | 436.35 (378.91 to 496.06) | 0.14 (-1.13 to 1.42) |
| Netherlands | 93458.39 (89628.04 to 96622.49) | 478.93 (460.21 to 494.9) | 142372.2 (130386.73 to 153821.48) | 435.34 (402.27 to 469.36) | -0.4 (-0.61 to -0.19) |
| Denmark | 40444.37 (39069.17 to 41776.8) | 522.47 (504.79 to 538.79) | 47754.81 (43566.28 to 51734.41) | 431.1 (395.7 to 464.94) | -0.62 (-0.94 to -0.29) |
| Portugal | 61985.94 (59819 to 64043.39) | 459.65 (442.95 to 474.55) | 92569.01 (85463.7 to 99117.98) | 421.32 (391.75 to 449.51) | -0.29 (-0.51 to -0.08) |
| San Marino | 149.16 (128.23 to 173.81) | 455.55 (391.14 to 531.33) | 255.76 (172.08 to 363.41) | 413.51 (272.75 to 603.31) | -0.31 (-0.39 to -0.24) |
| Russian Federation | 724298.5 (695937.66 to 750334.66) | 396.96 (380.59 to 411.51) | 939797.72 (822797.91 to 1069391.68) | 408.98 (357.72 to 465.96) | 0.33 (-0.66 to 1.34) |
| Slovenia | 11469.54 (8847.11 to 14832.05) | 471 (363.6 to 610.48) | 16809.15 (13087.89 to 21737.48) | 408.2 (317.46 to 532.11) | -0.94 (-1.34 to -0.53) |
| Norway | 30893.88 (29686.14 to 31745.38) | 479.39 (463.47 to 492.46) | 36431.98 (33543.92 to 38790.41) | 393.9 (364.53 to 418.86) | -0.72 (-0.97 to -0.46) |
| Belarus | 56226.38 (54237.65 to 58379.18) | 436.77 (421.42 to 453.2) | 60383.78 (47969.61 to 78137.16) | 388.65 (308.85 to 500.2) | -0.37 (-1.08 to 0.35) |
| Spain | 212515.47 (203254.23 to 220022.25) | 402.81 (385.8 to 416.61) | 350228.59 (320236.7 to 377643.3) | 387.26 (355.22 to 416.37) | -0.17 (-0.31 to -0.02) |
| Estonia | 9207.13 (8786.97 to 9627.4) | 452.83 (432.31 to 473.54) | 9664.72 (7667 to 12204.86) | 384.18 (304.24 to 486.56) | -0.63 (-1.31 to 0.06) |
| Latvia | 15002.61 (14399.77 to 15619.49) | 424.51 (407.56 to 441.98) | 14161.75 (11809.63 to 17164.57) | 377.36 (314.55 to 460.4) | -0.35 (-1.31 to 0.61) |
| Montenegro | 2047.21 (1728.67 to 2339.41) | 323.67 (273.46 to 368.56) | 3663.37 (3013.94 to 4386.29) | 377.31 (309.51 to 451.69) | 0.57 (0.31 to 0.83) |
| Lithuania | 17571.33 (16937.2 to 18299.64) | 393.85 (379.91 to 409.98) | 19672.78 (16149.33 to 23901.04) | 365.57 (299.13 to 446.11) | -0.18 (-0.76 to 0.41) |
| United Kingdom | 444018.93 (430459.63 to 453696.05) | 511.85 (497.49 to 522.46) | 433584.69 (409710.16 to 451673.76) | 365.24 (347.77 to 379.1) | -1.16 (-1.27 to -1.04) |
| Ireland | 21737.37 (20898.94 to 22576.61) | 545.58 (524.66 to 566.66) | 26561.93 (24282.72 to 28836.47) | 362.73 (332.19 to 392.64) | -1.4 (-1.61 to -1.19) |
| Germany | 625807.33 (601663.78 to 646368.61) | 505.56 (486.9 to 521.06) | 647921.02 (598987.32 to 694996.48) | 360.72 (336.71 to 385.91) | -1.18 (-1.5 to -0.87) |
| Belgium | 75019.71 (71683.58 to 77747.39) | 499.39 (477.6 to 517.93) | 73674.41 (67680.74 to 79242.02) | 337.46 (312.89 to 361.05) | -1.37 (-1.73 to -1.01) |
| Italy | 360137.69 (349208.76 to 368567.99) | 418.41 (405.66 to 427.91) | 436750.2 (402100.2 to 459967.4) | 330.96 (309.6 to 347) | -0.82 (-0.97 to -0.68) |
| France | 381365.85 (364683.61 to 395730.46) | 474.28 (454.79 to 490.76) | 422215.01 (381664.2 to 458064.39) | 328.65 (300.66 to 354.38) | -1.37 (-1.64 to -1.1) |
| Israel | 20929.37 (19936.34 to 21869.34) | 436.85 (415.07 to 455.84) | 36383.51 (33302.17 to 39184.86) | 322.98 (296.74 to 346.83) | -1.06 (-1.28 to -0.83) |
| Sweden | 53991.08 (51377.46 to 56128.11) | 381.25 (365.11 to 395.48) | 63293.37 (59047.1 to 67074.93) | 322.73 (303.51 to 341.26) | -0.61 (-0.76 to -0.46) |
| Luxembourg | 2950.27 (2793.9 to 3111.83) | 550.17 (521.28 to 579.57) | 3075.37 (2664.6 to 3506.41) | 313.45 (273.07 to 358.22) | -1.93 (-2.16 to -1.7) |
| Greece | 42386.06 (40404.95 to 44087.74) | 283.09 (269.83 to 294.08) | 66397.4 (60981.59 to 71241.06) | 297.9 (277.05 to 318.58) | 0.13 (-0.08 to 0.34) |
| Malta | 1491.36 (1396.52 to 1587.27) | 349.5 (327.12 to 371.56) | 2546.78 (2253.88 to 2880.43) | 289.73 (256.51 to 327.33) | -0.61 (-0.81 to -0.41) |
| Austria | 66622.26 (64172.06 to 68809.07) | 587.75 (566.7 to 607.44) | 46853.56 (43157.71 to 50276.64) | 275.36 (254.23 to 294.83) | -2.59 (-2.84 to -2.35) |
| Cyprus | 2069.09 (1848.35 to 2319.3) | 259.44 (231.72 to 290.02) | 5205.01 (4554.67 to 5928.18) | 273.58 (239.23 to 311.54) | 0.15 (-0.06 to 0.36) |
| Finland | 21398.08 (20453.53 to 22146.57) | 306.45 (292.9 to 316.99) | 28875.9 (26508.78 to 31339.14) | 253.56 (233.43 to 275.07) | -0.72 (-0.78 to -0.66) |
| Switzerland | 30167.77 (28802.1 to 31393.84) | 301.77 (289.15 to 313.46) | 40284.97 (36399.83 to 43854.79) | 244.77 (222.34 to 265.09) | -0.8 (-0.94 to -0.65) |
| Iceland | 865.41 (805.74 to 925.55) | 309.2 (287.88 to 330.27) | 1291 (1158.72 to 1435.68) | 241.2 (216.54 to 267.52) | -0.85 (-1.1 to -0.61) |
| Albania | 4476.63 (4186.62 to 4783.55) | 198.19 (184.32 to 212.03) | 8224.82 (6122.18 to 10807.93) | 204.54 (153.05 to 268.24) | 0.04 (-0.53 to 0.61) |

**Supplementary Table 3** CRC mortality in 44 European countries in 1990 and 2019 and AAPC from 1990 to 2019

| Mortality |  |  |  |  |  |
| --- | --- | --- | --- | --- | --- |
| location | 1990 |  | 2019 |  | 1990-2019 |
|  | Death cases NO.(95%UI) | ASR/100,000 (95% UI) | Death cases NO.(95%UI) | ASR/100,000 (95% UI) | AAPC (95%CI) |
| Hungary | 4421.13 (4239.38 to 4587.42) | 30.7 (29.38 to 31.89) | 5646.02 (4698.99 to 6699.7) | 28.56 (23.65 to 34.03) | -0.29 (-0.46 to -0.12) |
| Slovakia | 1410.54 (1339.84 to 1478.71) | 23.75 (22.58 to 24.88) | 2432.6 (1932.43 to 3037.77) | 26.31 (20.96 to 32.8) | 0.33 (0 to 0.65) |
| Serbia | 2275.11 (1842.73 to 2539.35) | 21.09 (16.95 to 23.71) | 4018.3 (3269.14 to 4942.52) | 25.38 (20.63 to 31.01) | 0.64 (0.38 to 0.9) |
| Croatia | 1386.53 (1304.11 to 1471.55) | 22.69 (21.28 to 24.04) | 2308.47 (1842.91 to 2847.48) | 25.28 (20.14 to 31.22) | 0.63 (-0.14 to 1.4) |
| Bulgaria | 2366.46 (2246.69 to 2484.03) | 19.4 (18.4 to 20.31) | 3671.6 (2991.28 to 4477.36) | 25.11 (20.41 to 30.62) | 0.89 (0.36 to 1.42) |
| Poland | 9180.43 (8820.66 to 9403.78) | 21.44 (20.51 to 22) | 17767.86 (15128.74 to 20757.73) | 24.72 (20.98 to 28.96) | 0.45 (0.34 to 0.56) |
| Monaco | 17.09 (13.62 to 20.56) | 22.69 (18.18 to 27.39) | 25.37 (20.68 to 29.89) | 24.27 (19.64 to 28.78) | 0.23 (0.19 to 0.28) |
| Andorra | 14.15 (11.1 to 18.88) | 28.75 (22.82 to 37.96) | 33.66 (26.28 to 42.23) | 22.97 (17.88 to 28.86) | -0.78 (-0.85 to -0.7) |
| Bosnia and Herzegovina | 543.67 (515.06 to 576.46) | 14.4 (13.54 to 15.31) | 1318.83 (1044.25 to 1638.71) | 22.32 (17.81 to 27.66) | 1.41 (1.09 to 1.74) |
| North Macedonia | 269.71 (246.28 to 290.1) | 15.05 (13.77 to 16.19) | 682.87 (544.78 to 849.81) | 22.13 (17.8 to 27.27) | 1.35 (1.2 to 1.51) |
| Denmark | 2045.97 (1947.44 to 2121.78) | 24.5 (23.43 to 25.37) | 2646.84 (2395.73 to 2871.78) | 21.75 (19.76 to 23.49) | -0.4 (-0.64 to -0.17) |
| Netherlands | 4657.96 (4377.58 to 4845.56) | 23.05 (21.68 to 23.95) | 7774.38 (7036.41 to 8443.66) | 21.75 (19.8 to 23.55) | -0.25 (-0.46 to -0.04) |
| Czechia | 4644.16 (4474.83 to 4784.55) | 33.76 (32.41 to 34.8) | 4677.62 (3863.77 to 5626.32) | 21.58 (17.8 to 25.98) | -1.82 (-2.31 to -1.33) |
| San Marino | 7.81 (6.71 to 9.02) | 23.63 (20.36 to 27.36) | 15.31 (10.55 to 20.91) | 21.24 (14.31 to 29.61) | -0.36 (-0.47 to -0.25) |
| Romania | 3548.45 (3417.68 to 3674.27) | 12.86 (12.39 to 13.31) | 7577.04 (6280.55 to 9022.07) | 20.18 (16.61 to 24.11) | 1.77 (1.3 to 2.25) |
| Portugal | 2966.43 (2838.58 to 3079.77) | 22.17 (21.13 to 23.03) | 5189.14 (4699.9 to 5585.54) | 20.11 (18.52 to 21.52) | -0.32 (-0.52 to -0.12) |
| Norway | 1611.37 (1518.52 to 1664.2) | 22.69 (21.47 to 23.4) | 2034.99 (1837.87 to 2176.11) | 19.84 (18.04 to 21.11) | -0.47 (-0.76 to -0.17) |
| Slovenia | 525.52 (412.86 to 662.46) | 21.69 (17.07 to 27.36) | 912.94 (719.85 to 1163.3) | 19.84 (15.58 to 25.45) | -0.65 (-0.99 to -0.3) |
| Ukraine | 15302.81 (14792.77 to 15835.54) | 21.46 (20.74 to 22.22) | 14581.72 (12548.23 to 16939.03) | 19.18 (16.46 to 22.37) | -0.38 (-0.93 to 0.17) |
| Spain | 10285.28 (9692.52 to 10728.9) | 18.95 (17.84 to 19.77) | 20011.12 (17767.76 to 21745.72) | 18.85 (17.05 to 20.21) | -0.05 (-0.28 to 0.17) |
| Estonia | 396.06 (377.53 to 413.51) | 19.42 (18.49 to 20.27) | 527.97 (424.64 to 660.09) | 18.51 (14.81 to 23.22) | -0.23 (-0.87 to 0.42) |
| Russian Federation | 29696.31 (28698.59 to 30770.67) | 16.71 (16.11 to 17.33) | 42834.42 (37637.48 to 48395.33) | 18.17 (15.96 to 20.54) | 0.47 (-0.34 to 1.28) |
| United Kingdom | 22538.26 (21499.62 to 23104.42) | 24.49 (23.35 to 25.09) | 24219.75 (22210.04 to 25338.48) | 18.09 (16.77 to 18.85) | -1.06 (-1.18 to -0.94) |
| Germany | 31780.05 (30043.24 to 33032.25) | 24.4 (23.04 to 25.31) | 37551.54 (34130.67 to 40326.31) | 18.01 (16.54 to 19.26) | -1.05 (-1.44 to -0.66) |
| Ireland | 1035.49 (991.33 to 1075.65) | 25.68 (24.49 to 26.65) | 1377.03 (1241.75 to 1497.68) | 18.01 (16.26 to 19.51) | -1.22 (-1.39 to -1.05) |
| Republic of Moldova | 797.9 (766.41 to 833.52) | 18.1 (17.38 to 18.9) | 1027.59 (896.57 to 1163.98) | 17.66 (15.45 to 20.01) | 0.28 (-0.86 to 1.44) |
| Latvia | 643.09 (616.33 to 667.92) | 17.98 (17.21 to 18.68) | 745.56 (625.83 to 891.45) | 17.63 (14.77 to 21.21) | 0.12 (-0.76 to 1) |
| Montenegro | 85.68 (72.39 to 97.71) | 14.21 (12.03 to 16.2) | 168.06 (137.42 to 200.16) | 17.23 (14.08 to 20.4) | 0.79 (0.53 to 1.06) |
| Belgium | 3997.87 (3747.11 to 4183.14) | 25.61 (23.99 to 26.79) | 4324.83 (3879.72 to 4686.77) | 17.1 (15.59 to 18.44) | -1.42 (-1.78 to -1.05) |
| Belarus | 2246.06 (2156.7 to 2335.33) | 17.47 (16.78 to 18.15) | 2734.02 (2230.37 to 3444.61) | 17.03 (13.86 to 21.48) | 0.02 (-0.65 to 0.68) |
| Lithuania | 746.05 (714.55 to 780.24) | 16.62 (15.9 to 17.38) | 1024.4 (857.04 to 1220.52) | 16.9 (14.05 to 20.27) | 0.14 (-0.51 to 0.8) |
| Israel | 1036.01 (976.25 to 1085.06) | 22.01 (20.69 to 23.07) | 2007.88 (1796.58 to 2172.02) | 16.56 (14.91 to 17.85) | -1.01 (-1.3 to -0.71) |
| France | 20261.27 (19037.14 to 21245.52) | 23.53 (22.15 to 24.58) | 25496.72 (22330.21 to 27995.55) | 16.41 (14.69 to 17.82) | -1.25 (-1.5 to -0.99) |
| Luxembourg | 146.92 (137.81 to 155.6) | 27 (25.25 to 28.53) | 169.51 (146.57 to 192.12) | 15.97 (13.87 to 18.08) | -1.81 (-2.05 to -1.56) |
| Sweden | 2854.34 (2672.2 to 2982.99) | 18.2 (17.12 to 18.95) | 3623.37 (3273.29 to 3862.99) | 15.96 (14.67 to 16.96) | -0.46 (-0.57 to -0.35) |
| Italy | 17359.63 (16678.44 to 17759.43) | 19.47 (18.63 to 19.95) | 24877.29 (22016.36 to 26457.11) | 15.83 (14.41 to 16.7) | -0.73 (-0.91 to -0.56) |
| Greece | 2132.23 (2016.52 to 2227.09) | 14.19 (13.35 to 14.86) | 4000.11 (3599.73 to 4304.91) | 14.87 (13.67 to 15.87) | 0.14 (-0.13 to 0.41) |
| Cyprus | 95.95 (84.48 to 108.98) | 13.28 (11.7 to 15.1) | 265.29 (229.77 to 301.34) | 14.32 (12.4 to 16.32) | 0.23 (-0.03 to 0.49) |
| Malta | 70.49 (65.49 to 75.05) | 17 (15.79 to 18.12) | 136.44 (119.54 to 154.43) | 14.08 (12.38 to 15.89) | -0.63 (-0.91 to -0.34) |
| Austria | 3276.45 (3116.67 to 3388.43) | 27.16 (25.84 to 28.08) | 2603.19 (2355.01 to 2811.01) | 13.54 (12.36 to 14.54) | -2.38 (-2.61 to -2.16) |
| Finland | 1050.13 (995.04 to 1092.68) | 14.62 (13.81 to 15.22) | 1615.71 (1455.4 to 1756.19) | 12.33 (11.25 to 13.35) | -0.59 (-0.7 to -0.47) |
| Switzerland | 1512.82 (1424.49 to 1577.97) | 14.06 (13.29 to 14.64) | 2234.27 (1979.68 to 2431.3) | 11.85 (10.7 to 12.84) | -0.65 (-0.84 to -0.46) |
| Iceland | 43.08 (39.62 to 46.17) | 14.7 (13.54 to 15.74) | 69.79 (60.81 to 78.21) | 11.81 (10.53 to 13.16) | -0.76 (-1.07 to -0.44) |
| Albania | 169.92 (157.35 to 182.06) | 8.63 (7.94 to 9.26) | 385.19 (290.5 to 501.54) | 9.15 (6.96 to 11.89) | 0.16 (-0.34 to 0.66) |
